# Supplementary material for: Water, sanitation, and depressive symptoms in Indonesia: The mediating role of life satisfaction
Source: PLoS One. 2026 Feb 5;21(2):e0341886. doi: 10.1371/journal.pone.0341886 (PMC12875457; doi:10.1371/journal.pone.0341886)
Supplement: S1 Table — (DOCX) [file pone.0341886.s001.docx]

**S1 Table. Complete Sanitation and Water Safety Classification Table**

| **Variable** | **Question asked** | **Categories** | **Improved/Safe (0)** | **Unimproved/Unsafe (1)** |
| --- | --- | --- | --- | --- |
| Drinking Water Sources | What is the main source of drinking water for this household? | Piped private, piped to yard/plot, unprotected tube well, unprotected spring, rainwater collection, surface water, unprotected pond, delivered by tanker-truck, bottled mineral water, other unspecified sources. | Piped private, piped to yard/plot, protected spring, rainwater collection, bottled mineral water, boiled water. | Unprotected tube wells, surface water, unprotected ponds, tanker-truck/lorry, other unspecified sources. |
| Water Source (Household Activities) | Where does this household get most of its water for other purposes, such as bathing and washing clothes? | Piped water in household, piped to yard/plot, unprotected tube well, unprotected spring, rainwater collection, surface water, unprotected pond, stored in a tank, other unspecified sources. | Piped water within household, piped to yard/plot, unprotected tube wells, unprotected springs, rainwater collection. | Surface water, unprotected ponds, other unspecified sources. |
| Toilet Facilities | Where do most household members defecate? | Flush/pour into septic tank, flush/pour into pit latrine, shared flush toilet, public flush toilet, open defecation into rivers, open defecation into gardens, open defecation into sewers, open defecation into ponds, open defecation into cattle pens, open defecation into oceans/rivers, other unspecified. | Flush or pour into septic tank. | All other responses (open defecation or unspecified locations). |
| Liquid Waste Disposal | How does this household dispose of wastewater (e.g., used water from washing and bathing)? | Flowing sewers, non-flowing sewers, permanent pits, rivers, dumped in yard/garden, ponds, puddles, rice fields, oceans, other unspecified. | Flowing sewers, non-flowing sewers/gutters, permanent pits. | Dumped in rivers, yard/garden, ponds, puddles, rice fields, oceans, or other unspecified methods. |
| Household Waste Disposal | How does this household dispose of rubbish? | Rubbish bin collected by officers, burned, dumped into rivers, thrown in yard/garden, buried in a hole, forest, sea/lake/beach, rice fields, or other unspecified methods. | Rubbish bin collected by waste management officers | All other methods (burned, dumped, thrown in yard/garden, buried). |
