# Supplementary material for: Water, sanitation, and depressive symptoms in Indonesia: The mediating role of life satisfaction
Source: PLoS One. 2026 Feb 5;21(2):e0341886. doi: 10.1371/journal.pone.0341886 (PMC12875457; doi:10.1371/journal.pone.0341886)
Supplement: S2 Table — (DOCX) [file pone.0341886.s002.docx]

**S2 Table. Correlation Matrix**

|  | **Safe Drinking Water** | **Improved Water Source** | **Improved Toilet Facility** | **Safe Liquid Waste Disposal** | **Safe Waste Disposal** | **Depression** |
| --- | --- | --- | --- | --- | --- | --- |
| **Safe Drinking Water** | 1.00 |  |  |  |  |  |
| **Improved Water Source** | 0.4112 | 1.00 |  |  |  |  |
| **Improved Toilet Facility** | 0.0952 | 0.2047 | 1.00 |  |  |  |
| **Safe Liquid Waste Disposal** | 0.1120 | 0.2055 | 0.2604 | 1.00 |  |  |
| **Safe Waste Disposal** | 0.0562 | 0.1107 | 0.1733 | 0.2997 | 1.00 |  |
| **Depression** | 0.0241 | 0.0249 | 0.0415 | 0.0248 | 0.0163 | 1.00 |
