# Supplementary material for: Water, sanitation, and depressive symptoms in Indonesia: The mediating role of life satisfaction
Source: PLoS One. 2026 Feb 5;21(2):e0341886. doi: 10.1371/journal.pone.0341886 (PMC12875457; doi:10.1371/journal.pone.0341886)
Supplement: S3 Table — (DOCX) [file pone.0341886.s003.docx]

**S3 Table.** **Overall goodness-of-fit statistics for the sensitivity analyses**

| **Fit Index** | **Fit statistic indices** | | | |
| --- | --- | --- | --- | --- |
|  | SRMR | RMSEA | CFI | NNFI (TLI) |
| **Drinking water** | 0.019 | 0.039 | 0.755 | 0.484 |
| **Water source** | 0.019 | 0.038 | 0.814 | 0.609 |
| **Toilet facilities** | 0.020 | 0.046 | 0.848 | 0.682 |
| **Sewage disposal method** | 0.018 | 0.037 | 0.903 | 0.795 |
| **Waste disposal method** | 0.016 | 0.035 | 0.967 | 0.931 |
