# Supplementary material for: Water, sanitation, and depressive symptoms in Indonesia: The mediating role of life satisfaction
Source: PLoS One. 2026 Feb 5;21(2):e0341886. doi: 10.1371/journal.pone.0341886 (PMC12875457; doi:10.1371/journal.pone.0341886)
Supplement: S4 Table — (DOCX) [file pone.0341886.s004.docx]

**S4 Table. Overall goodness-of-fit statistics of each model**

| **Fit Index** | **Model A** | | | | **Model B** | | | |
| --- | --- | --- | --- | --- | --- | --- | --- | --- |
|  | SRMR | RMSEA | CFI | NNFI (TLI) | SRMR | RMSEA | CFI | NNFI (TLI) |
| **Drinking water** | 0.004 | 0.008 | 0.987 | 0.970 | 0.008 | 0.018 | 0.959 | 0.897 |
| **Water source** | 0.005 | 0.011 | 0.990 | 0.979 | 0.007 | 0.016 | 0.973 | 0.932 |
| **Toilet facilities** | 0.005 | 0.013 | 0.985 | 0.967 | 0.007 | 0.016 | 0.979 | 0.947 |
| **Sewage disposal method** | 0.006 | 0.014 | 0.987 | 0.971 | 0.006 | 0.015 | 0.987 | 0.968 |
| **Waste disposal method** | 0.007 | 0.020 | 0.994 | 0.987 | 0.007 | 0.019 | 0.993 | 0.983 |
