# Supplementary material for: Water, sanitation, and depressive symptoms in Indonesia: The mediating role of life satisfaction
Source: PLoS One. 2026 Feb 5;21(2):e0341886. doi: 10.1371/journal.pone.0341886 (PMC12875457; doi:10.1371/journal.pone.0341886)
Supplement: S5 Table — (DOCX) [file pone.0341886.s005.docx]

**S5 Table. Combinations of Water and Sanitation Issues**

| **Combination** | **Drinking Water Source** | **Water Source (Household)** | **Toilet Facility** | **Liquid Waste Disposal** | **Waste Disposal** | **n** | **%** |
| --- | --- | --- | --- | --- | --- | --- | --- |
| **1** | Improved | Improved | Improved | Safe | Safe | 8,573 | 27.30 |
| **2** | Improved | Improved | Improved | Safe | Unsafe | 9,618 | 30.63 |
| **3** | Improved | Improved | Improved | Unsafe | Safe | 741 | 2.36 |
| **4** | Improved | Improved | Improved | Unsafe | Unsafe | 3,954 | 12.59 |
| **5** | Improved | Improved | Unimproved | Safe | Safe | 1,356 | 4.32 |
| **6** | Improved | Improved | Unimproved | Safe | Unsafe | 2,591 | 8.25 |
| **7** | Improved | Improved | Unimproved | Unsafe | Safe | 255 | 0.81 |
| **8** | Improved | Improved | Unimproved | Unsafe | Unsafe | 2,745 | 8.74 |
| **9** | Improved | Unimproved | Improved | Safe | Safe | 65 | 0.21 |
| **10** | Improved | Unimproved | Improved | Safe | Unsafe | 177 | 0.56 |
| **11** | Improved | Unimproved | Improved | Unsafe | Safe | 28 | 0.09 |
| **12** | Improved | Unimproved | Improved | Unsafe | Unsafe | 151 | 0.48 |
| **13** | Improved | Unimproved | Unimproved | Safe | Safe | 18 | 0.06 |
| **14** | Improved | Unimproved | Unimproved | Safe | Unsafe | 144 | 0.46 |
| **15** | Improved | Unimproved | Unimproved | Unsafe | Safe | 37 | 0.12 |
| **16** | Improved | Unimproved | Unimproved | Unsafe | Unsafe | 571 | 1.82 |
| **17** | Unimproved | Improved | Improved | Safe | Safe | 8 | 0.03 |
| **18** | Unimproved | Improved | Improved | Safe | Unsafe | 22 | 0.07 |
| **19** | Unimproved | Improved | Improved | Unsafe | Safe | 1 | 0.01 |
| **20** | Unimproved | Improved | Improved | Unsafe | Unsafe | 1 | 0.01 |
| **21** | Unimproved | Improved | Unimproved | Safe | Safe | 0 | 0 |
| **22** | Unimproved | Improved | Unimproved | Safe | Unsafe | 5 | 0.02 |
| **23** | Unimproved | Improved | Unimproved | Unsafe | Safe | 2 | 0.01 |
| **24** | Unimproved | Improved | Unimproved | Unsafe | Unsafe | 20 | 0.06 |
| **25** | Unimproved | Unimproved | Improved | Safe | Safe | 10 | 0.03 |
| **26** | Unimproved | Unimproved | Improved | Safe | Unsafe | 29 | 0.09 |
| **27** | Unimproved | Unimproved | Improved | Unsafe | Safe | 5 | 0.02 |
| **28** | Unimproved | Unimproved | Improved | Unsafe | Unsafe | 65 | 0.21 |
| **29** | Unimproved | Unimproved | Unimproved | Safe | Safe | 3 | 0.01 |
| **30** | Unimproved | Unimproved | Unimproved | Safe | Unsafe | 24 | 0.08 |
| **31** | Unimproved | Unimproved | Unimproved | Unsafe | Safe | 13 | 0.04 |
| **32** | Unimproved | Unimproved | Unimproved | Unsafe | Unsafe | 171 | 0.54 |
