# Supplementary material for: Water, sanitation, and depressive symptoms in Indonesia: The mediating role of life satisfaction
Source: PLoS One. 2026 Feb 5;21(2):e0341886. doi: 10.1371/journal.pone.0341886 (PMC12875457; doi:10.1371/journal.pone.0341886)
Supplement: S6 Table — (DOCX) [file pone.0341886.s006.docx]

**S6 Table. Cumulative Sanitation Issues and Depression**

| **Number of Sanitation Issues** | **Description** | **n** | **% of Total Sample** | **Depression Cases (n)** | **% Depression** |
| --- | --- | --- | --- | --- | --- |
| 0 | No issues (all sanitation variables are improved/safe) | 8,573 | 27.30 | 1,813 | 21 |
| 1 | 1 issue (one unimproved/unsafe sanitation condition) | 11,788 | 37.54 | 2,700 | 22 |
| 2 | 2 issues (two unimproved/unsafe sanitation conditions) | 7,056 | 22.47 | 1,731 | 24 |
| 3 | 3 issues (three unimproved/unsafe sanitation conditions) | 3,122 | 9.94 | 810 | 26 |
| 4 | 4 issues (four unimproved/unsafe sanitation conditions) | 693 | 2.21 | 189 | 27 |
| 5 | 5 issues (all sanitation variables are unimproved/unsafe) | 171 | 0.54 | 58 | 34 |
