# Supplementary material for: Water, sanitation, and depressive symptoms in Indonesia: The mediating role of life satisfaction
Source: PLoS One. 2026 Feb 5;21(2):e0341886. doi: 10.1371/journal.pone.0341886 (PMC12875457; doi:10.1371/journal.pone.0341886)
Supplement: S7 Table — (DOCX) [file pone.0341886.s007.docx]

**S7 Table. Logistic regression results on the associations between each of the risk factors and depression among Indonesian adults**

| **Variables** | **Model 1** | **Model 2** | **Model 3** | **Model 4** | **Model 5** |
| --- | --- | --- | --- | --- | --- |
|  | **OR (95% CI)** | **OR (95% CI)** | **OR (95% CI)** | **OR (95% CI)** | **OR (95% CI)** |
| Drinking water (ref: unsafe) | 1.57 (1.24 – 2.00)^*^ |  |  |  |  |
| Water source (ref: unimproved) |  | 1.25 (1.10 – 1.41)^*^ |  |  |  |
| Toilet facility (ref: unimproved) |  |  | 1.17 (1.10 – 1.24)^*^ |  |  |
| Sewage disposal (ref: unimproved) |  |  |  | 1.13 (1.06 – 1.20)^*^ |  |
| Waste disposal (ref: unimproved) |  |  |  |  | 1.09 (1.02 – 1.16)^+^ |
| Urban residence | 1.07 (1.01 – 1.14)^+^ | 1.08 (1.02 – 1.14)^+^ | 1.08 (1.02 – 1.15)^#^ | 1.09 (1.03 – 1.16)^#^ | 1.11 (1.04 – 1.18)^#^ |
|  |  |  |  |  |  |
| Age (years), mean (SD) | 0.97 (0.97 – 0.98)^+^ | 0.97 (0.97 – 0.98)^+^ | 0.97 (0.97 – 0.98)^*^ | 0.97 (0.97 – 0.98)^*^ | 0.97 (0.97 – 0.98)^*^ |
|  |  |  |  |  |  |
| Female Gender (ref: male) | 1.07 (1.01 – 1.14)^+^ | 1.08 (1.02 – 1.14)^+^ | 1.08 (1.02 – 1.14)^+^ | 1.07 (1.01 – 1.14)^+^ | 1.07 (1.01 – 1.14)^+^ |
|  |  |  |  |  |  |
| Employed (ref: unemployed) | 1.06 (1.00 – 1.13)^+^ | 1.06 (1.00 – 1.13)^+^ | 1.06 (1.00 – 1.12)^+^ | 1.06 (1.00 – 1.13)^+^ | 1.06 (1.00 – 1.13)^+^ |
|  |  |  |  |  |  |
| Education (ref: primary) |  |  |  |  |  |
| Secondary | 0.77 (0.72 – 0.82)^*^ | 0.77 (0.72 – 0.83)^*^ | 0.79 (0.74 – 0.84)^*^ | 0.78 (0.72 – 0.83)^*^ | 0.77 (0.72 – 0.83)^*^ |
| College | 0.67 (0.61 – 0.74)^*^ | 0.68 (0.61 – 0.74)^*^ | 0.70 (0.63 – 0.77)^*^ | 0.68 (0.62 – 0.75)^*^ | 0.68 (0.62 – 0.75)^*^ |
| Log Likelihood | -16212.539 | -16213.029 | -16207.285 | -16211.860 | -16215.624 |
| Nagelkerke Pseudo-R2 | 0.012 | 0.012 | 0.013 | 0.014 | 0.021 |
| Hosmer-Lemeshow Chi2 | 23.91 | 21.62 | 10.09 | 11.91 | 17.73 |
| Observations | 30,176 | 30,176 | 30,176 | 30,176 | 30,176 |

Note: OR=Odds Ratio; CI =Confidence Interval in parenthesis; ^+^p<0.05, ^#^<0.005, ^*^p<0.001
