# Supplementary material for: Water, sanitation, and depressive symptoms in Indonesia: The mediating role of life satisfaction
Source: PLoS One. 2026 Feb 5;21(2):e0341886. doi: 10.1371/journal.pone.0341886 (PMC12875457; doi:10.1371/journal.pone.0341886)
Supplement: S8 Table — (DOCX) [file pone.0341886.s008.docx]

**S8 Table. Standardised Coefficients for Direct Effects of Sanitation on Depression (Model A) and Indirect Effects via LS as a Mediator (Model B), SEM Analysis among the Two Lowest Wealth Quintiles (N= 11,622)**

| **Sanitation Variables** | **Direct – Model A** | **Indirect – Model B** | | |
| --- | --- | --- | --- | --- |
|  | **Depression** | **Sanitation 🡪 Depression** | **Sanitation 🡪 LS** | **LS 🡪 Depression** |
| Drinking water | 0.028 (0.010 – 0.046)^#^ | 0.026 (0.008 – 0.044)^#^ | 0.012 (-0.005 – 0.030) | 0.124 (-0.005 – 0.030) |
| Water source | 0.020 (0.001 – 0.038)^+^ | 0.016 (-0.001 – 0.034) | 0.026 (0.008 – 0.044)^#^ | 0.122 (0.104 – 0.140)^*^ |
| Toilet facilities | 0.021 (0.003 – 0.039)^+^ | 0.122 (0.104 – 0.140)^*^ | 0.047 (0.029 – 0.065)^*^ | 0.122 (0.104 – 0.140)^*^ |
| Sewage disposal method | 0.008 (-0.009 – 0.026) | 0.006 (-0.011 – 0.24) | 0.016 (-0.013 – 0.034) | 0.123 (0.105 – 0.141)^*^ |
| Waste disposal method | 0.003 (-0.014 – 0.021) | 0.001 (-0.016 – 0.019) | 0.014 (-0.035 – 0.032) | 0.123 (-0.016 – 0.019) |

Note: Standardized coefficient; ^+^p<0.05, ^#^<0.005, ^*^p<0.001
