# Supplementary material for: Water, sanitation, and depressive symptoms in Indonesia: The mediating role of life satisfaction
Source: PLoS One. 2026 Feb 5;21(2):e0341886. doi: 10.1371/journal.pone.0341886 (PMC12875457; doi:10.1371/journal.pone.0341886)
Supplement: Attachment — Submitted filename: PLOS ONE Reviewers.docx [file pone.0341886.s009.docx]

**Review**

**Water, Sanitation, and Depression in Indonesia: The Mediating Role of Life Satisfaction**

**Abstrac**t

Line 31: Please add the duration of observation. How long was the questionnaire data collected?

Line 36: Correct the numerical error: *(β=0.54)* → *(β=0.054).*

**Introduction**

Lines 63–69: The information presented is relevant to the title. However, paragraphs 2 and 3 appear to lack coherence. The connection between the discussion of depression in Indonesia (paragraph 2) and issues of water and sanitation (paragraph 3) is somewhat abrupt. It would be helpful to include data sources related to population size, water quality vulnerability, and sanitation health in Indonesia.

Lines 80–89: Is the explanation of the mediating role of life satisfaction (LS) in the model theoretically robust enough? The authors should clarify why LS was selected as the primary mediator instead of other social factors, such as social support or environmental stress.

**Method**

Table 1: For *liquid waste disposal*, the “safe” category lists discharge into rivers, while the “unsafe” category also includes discharge into rivers. The authors should clarify the distinction between these two classifications.

Lines 115–134: The explanation of the IFLS dataset is comprehensive; however, the access time (“November 2024”) appears inconsistent with the 2014–2015 data. This should be corrected to avoid the impression that the data were accessed after the analysis had been conducted.

Ensure consistent use of “Model 1” and “Model 2” or “Model A” and “Model B.” In the Methods section, the text refers to Models 1 and 2, whereas in the Analysis section, it switches to Models A and B.

**Result and Discussion**

The discussion remains overly descriptive and does not sufficiently emphasise the study’s novelty compared with previous research. The authors could add a concluding paragraph to Section 4.1 highlighting the contribution of this study relative to research conducted in Bangladesh or Ghana.

**Spelling and Sentence Structure**

1. Lines 231-232: Please use the present tense in the entire result sentence, e.g. "Table 2 presents..."
2. Lines 381-383: The sentence "WHO further estimated that every US$1 invested...", please change to "The WHO further estimates..." in order to parallel the academic style.
3. Use the term “life satisfaction (LS)” consistently throughout the manuscript. Some sections spell it out in full without the abbreviation.
4. Add a source note to each table (e.g., *Data source: IFLS-5, authors’ calculation*).
5. In British style, the use of a comma before *and* is not required unless necessary to prevent ambiguity.
6. Line 274: “Standardized coefficient” 🡪 “Standardised coefficient”
7. Line 384: “sanitation programs…” 🡪 “sanitation programmes…”
8. Please review and ensure consistency with British English conventions across the manuscript.
9. Improve sentence structure for clarity and conciseness, avoiding redundancy.

**References**

Please use the most recent references to support your research. References to numbers [25], [32], [44], [58], and [59] used over the last 10 years.

In example:

Sakti, B., Wibawa, S., Maharani, A. T., Andhikaputra, G., Savira, M., Putri, A., Iswara, A. P., Sapkota, A., Sharma, A., Syafei, A. D., & Wang, Y. (2023). Effects of Ambient Temperature , Relative Humidity , and Precipitation on Diarrhea Incidence in Surabaya. *International Journal of Environmental Research and Public Health*, *20*, 2313.

Wulandari, R., Iswara, A. P., Qadafi, M., Prayogo, W., Astuti, R. D. P., Utami, R. R., Jayanti, M., Awfa, D., Suryawan, I. W. K., Fitria, L., & Andhikaputra, G. (2024). Water pollution and sanitation in Indonesia: a review on water quality, health and environmental impacts, management, and future challenges. *Environmental Science and Pollution Research*, *0123456789*. https://doi.org/10.1007/s11356-024-35567-x
