# Supplementary material for: Water, sanitation, and depressive symptoms in Indonesia: The mediating role of life satisfaction
Source: PLoS One. 2026 Feb 5;21(2):e0341886. doi: 10.1371/journal.pone.0341886 (PMC12875457; doi:10.1371/journal.pone.0341886)
Supplement: Attachment — Submitted filename: Sanitation Paper Final Manuscript - SS Review.docx [file pone.0341886.s011.docx]

**Water, Sanitation, and Depression in Indonesia: The Mediating Role of Life Satisfaction**

Mashita Fajri^1^, Sri Idaiani^2^, Amy Blakemore^3^, Jonathan Gibson^4^, Herni Susanti^1^, Helen Brooks^3^, Penny Bee^3^, Asri Maharani^3*^

Affiliations:

1. Department of Mental Health Nursing, Faculty of Nursing, Universitas Indonesia, Depok, Indonesia.
2. Research Centre for Preclinical and Clinical Medicine, National Research and Innovation Agency, Indonesia.
3. Division of Nursing, Midwifery & Social Work, University of Manchester, Manchester, UK.
4. Centre for Primary Care and Health Services Research, University of Manchester, Manchester, UK.

* Corresponding Author:

Email address: asri.maharani@manchester.ac.uk

# Abstract

Access to clean water and adequate sanitation is vital for public health. However, the effects of water and sanitation on depression remain insufficiently explored among the general population in low- and middle-income countries (LMICs). This study aims to examine the links between sanitation conditions and depression, and to evaluate the mediating role of life satisfaction (LS) among adults in Indonesia. We used the fifth wave of the Indonesian Family Life Survey (IFLS-5) dataset with participants aged 18 and above (mean: 37.32 years; SD: 14.93), collected in 2014/2015. Depression was measured using the CES-D-10 scale. Five sanitation indicators were included: drinking water, water source, toilet facilities, liquid waste disposal, and safe waste disposal methods. Structural equation modelling (SEM) was employed to estimate the direct and indirect pathways linking sanitation factors and depression through LS, adjusting for age and sex. The study involved 31,446 participants, of whom 7,312 (23%) were classified as depressed. In the first SEM model, drinking water (β=0.023, p<0.001), water source (β=0.033, p<0.001), toilet facilities (β=0.039, p<0.001), and waste disposal method (β=0.021, p<0.005) were directly associated with depressive symptoms, while sewage disposal was not significant. In the second SEM model, which included LS as a mediator, the direct effects remained consistent: drinking water (β=0.013, p<0.05), water source (β=0.54, p<0.001), toilet facilities (β=0.068, p<0.001), and waste disposal method (β=0.046, p<0.001). Additionally, unimproved sanitation was significantly linked to lower LS, and LS was strongly associated with depression across all sanitation factors (β≈0.124–0.126, p<0.001). Poor sanitation, including unsafe drinking water, inadequate toilets, and improper waste disposal, is linked to a higher risk of depression among Indonesian adults, with life satisfaction as a partial mediator. Interventions should focus on improving access to safe drinking water and sanitation, as these are cost-effective ways to enhance mental health and provide substantial economic outputs.

# 1. Introduction

The global burden of mental health issues is significant, with nearly 1 billion people affected worldwide. Among these, depression and anxiety are the most prevalent, with 279.6 million (28.8%) and 301.4 million cases (31.1%) [1]. Over the past 30 years, the prevalence of both has increased significantly. Depression cases have increased by over 1.6 times, and anxiety cases have grown by about 1.5 times during this period [1]. This burden is especially pronounced in low- and middle-income countries (LMICs), where over 75% of individuals with mental disorders reside [2]. In these regions, mental health conditions contribute significantly to the overall disease burden, accounting for 16.6% of the overall disease burden in 2019. Limited resources, insufficient healthcare infrastructure, and poor access to mental health services in those countries exacerbate these challenges [3].

In Indonesia, a country with a large and diverse population, approximately 14 million people suffer from depression and anxiety, placing it among the top two countries in the WHO South-East Asia region for the prevalence of these disorders [4]. The impact of mental health issues extends across all aspects of life. Recent studies in Indonesia found that depression is known to reduce work retention by 5.5% [5] and significantly increase the risk of suicide among adults [6]. The financial burden associated with mental health disorders is substantial, with annual treatment costs for depression and anxiety estimated to range between $85 - $114 per patient. When aggregated on a national scale, these costs could total up to $6.244 billion, reflecting the substantial economic impact of mental health issues [7].

Understanding the link between water and sanitation-related factors and health is increasingly recognised as a crucial component of public health. Sanitation encompasses access to clean water, proper waste disposal, and hygienic living conditions, all of which are vital for both physical and mental well-being [8]. The absence of safe sanitation can lead to numerous adverse health outcomes, including diarrhoea, anaemia, and complications such as early pregnancy loss and pre-term birth [9]. The impact extends beyond physical health; the lack of basic sanitation amenities often results in psychological distress, anxiety, and depression, particularly among women and girls [9].

Evidence from low-income and marginalised communities highlights these impacts, with studies from Mozambique, Bangladesh, and India showing that inadequate or unsafe sanitation facilities contribute to fear, stress, and psychological distress [10-12]. Research from Kenya and Bangladesh further demonstrates that consistent toilet and latrine access improves psychosocial health and happiness [11, 13]. A systematic review supports these findings by highlighting the link between poor sanitation and mental health issues like anxiety and depression [14]. These challenges often arise from the constant fear and stress caused by unsafe sanitation facilities or having to resort to open defecation. The connection between poor health and limited access to essential services can also create a vicious cycle, where physical illness exacerbates mental health challenges [15], and poor mental health, in turn, worsens physical conditions [16].

Building on this evidence, psychosocial factors such as life satisfaction (LS) may play a mediating role in the sanitation–mental health pathway. LS is a key component of overall psychological well-being and has been shown to foster resilience and adaptive coping [17, 18]. Previous studies have shown that higher life satisfaction is positively associated with various aspects of subjective well-being [18] and resilience [17]. This mechanism aligns with the Transdisciplinary Neighbourhood Health Framework, developed using data from the Canadian Longitudinal Study on Aging, which posits that environmental conditions influence mental well-being through socio-behavioural mediators such as social support, loneliness, and LS, ultimately shaping depressive outcomes [19]. Applying this lens to sanitation suggests that improvements in environmental conditions may enhance LS, which in turn mitigates depression risk.

Despite this growing body of research, there remains a pressing need for comprehensive studies that explore the association between water, sanitation, and depression, as current research is limited in scope and geographic coverage. Most studies have been conducted in high-income countries like the USA [20] and Canada [21]. These targeted studies limit the generalisability of findings, as they may not reflect the experiences of broader populations in LMIC settings. Furthermore, similar studies conducted in LMICs (Bangladesh, Brazil, China, and Ghana) have focused on specific populations, such as women, older adults [22-24], or those living in slums [25]. This may limit the evidence base needed to inform equitable public health interventions and policy strategies addressing sanitation-related mental health risks.

In Indonesia, where disparities in access to clean water and sanitation facilities remain evident, with approximately 47% of the population lacking adequate sanitation and 22% lacking access to clean water [26], investigating the potential impact of these issues on mental health is essential. Such research is relevant where environmental inequities and vulnerabilities intersect, but empirical evidence linking infrastructure to psychological outcomes is scarce. Existing research on sanitation and mental health in Indonesia has primarily focused on sanitation workers [27], emotional disturbances rather than depression [28], and often involving small, non-representative samples of fewer than 100 participants. Beyond access to sanitation, cultural beliefs significantly influence sanitation behaviours. A study in rural Bali found that supernatural beliefs, viewing toilets as polluting, and prioritising religious spending over sanitation impacted toilet ownership and use [29]. Given the intertwined environmental, cultural, and social dimensions, this study aims to investigate the relationships between water, sanitation, and depression, with life satisfaction as a potential mediator in the Indonesian context.

# 2. Methods

## 2.1 Data and study population

The data used in this study were obtained from the fifth wave of the Indonesian Family Life Survey (IFLS-5), conducted between 2014 and 2015 [30]. The survey is jointly administered by the RAND Corporation (USA), the University of Gadjah Mada, and SurveyMETER (both located in Yogyakarta, Indonesia). Initiated in 1998, the IFLS-5 sample represents approximately 83% of the Indonesian population. IFLS-5 provides individual-level data for Indonesians aged 15 and older, including demographics, socio-economic status, sanitation, health, and depressive symptoms. The survey used a multistage stratified sampling strategy by province and urban–rural location, randomly selecting household members for detailed interviews [30]. Of the initial 50,000 participants, this study included adults (≥15 years) who completed the Health Conditions (KK), Household Characteristics (KR), Welfare (SW), and Psychological Health (KP) sections, resulting in 31,446 respondents after excluding those with missing data on chronic disease count or depressive symptoms. To ensure representativeness and to account for the complex sampling structure, all statistical analyses applied the individual-level sampling weights provided in IFLS-5. Access to the data is provided at RAND’s website [31], with the research team storing the files in a secure institutional Dropbox folder. Although the dataset had been available within the team’s shared storage, the authors first accessed the data for research purposes in November 2024. Ethical clearance for the IFLS was obtained from the Institutional Review Boards of RAND (USA) and the University of Gadjah Mada (Indonesia). All participants provided written informed consent prior to their participation. The dataset is fully anonymised, and no identifying information about individual participants was accessible to the authors during or after data analysis.

## 2.2 Measurement and variable definitions

### Depression

Depressive symptoms were evaluated using the 10-item Center for Epidemiologic Studies Depression Scale (CES-D-10), which assesses the frequency of feelings and behaviours over the past week [32]. Respondents rated each of the ten items on a four-point Likert scale: "rarely or none (≤1 day)", "some days (1–2 days)", "occasionally (3–4 days)", and "most of the time (5–7 days)". Scores for each item ranged from zero to four, with a total score ranging from 0 to 30. A lower total score indicates fewer depressive symptoms. The CES-D-10 has demonstrated strong validity and reliability among the Indonesian population [33]. Consistent with previous research using the IFLS, respondents were categorised as experiencing depression if their CES-D-10 score was 10 or higher [34]. This approach ensures continuity with prior studies and allows for meaningful comparisons across different populations and time periods.

### Sanitation Conditions

In the IFLS survey, participants were asked to report their primary sources of drinking water, water used for household activities (such as bathing and washing clothes), their toilet facilities, methods for disposing of liquid waste, and methods for disposing of household waste. While the WHO/UNICEF Joint Monitoring Programme (JMP) currently recommends a five-tier service ladder for sanitation facilities, including ‘safely managed,’ ‘basic,’ ‘limited,’ ‘unimproved,’ and ‘no service’ [32], not all IFLS survey responses provide sufficient detail to classify households according to these levels. The JMP ladders are primarily designed for international benchmarking and to capture incremental service quality across countries, building on the traditional classification of facilities as improved or unimproved [32]. However, because the IFLS questionnaire lacks several indicators required for the five-tier system and there are no published studies or policy documents applying the full JMP ladder to the Indonesian context, we were unable to operationalise the five-tier classification in this study.

Following previous studies that have used IFLS sanitation data [35], we dichotomised responses into “safe” versus “unsafe” for drinking water source and waste disposal (liquid and solid), and “improved” versus “unimproved” for household water sources and toilet facilities. For drinking water, we defined it as “safe” if it came from an improved source (pipe, pumped well, spring, rainwater, or bottled mineral water) and/or if the household reported boiling the water before consumption, and “unsafe” otherwise. This approach captures both the quality of the water source and household treatment practices, while also ensuring comparability with earlier research and a consistent understanding of the quality and safety of water, sanitation, and waste management practices across the study population. For toilet facilities, although more granular classifications exist, we followed the dichotomisation approach applied in prior research [36], which demonstrates that reducing toilet facilities into two categories is both analytically valid and widely accepted in public health studies. Table 1 outlines the various responses provided by participants and how these were grouped into categories based on the standard definitions of "improved" or "safe" (coded as 0) and "unimproved" or "unsafe" (coded as 1). **S1 Table** provides details about the survey questions for each variable.

**Table 1.** **Classifications of sanitation conditions.**

| **Variable** | **Improved/Safe (0)** | **Unimproved/Unsafe (1)** |
| --- | --- | --- |
| **Drinking Water Sources** | Pipe water, well/pump, spring water, rain water, mineral water, and those who report boiling the water before drinking. | Well without a pump, river/creek water, pond/fishpond, water collection basin. |
| **Water Source (Household Activities)** | Piped water within household, piped to yard/plot, unprotected tube wells, unprotected springs, rainwater collection. | Surface water, unprotected ponds, stored in a tank, and other unspecified sources. |
| **Toilet Facilities** | Flush or pour into septic tank. | Flush/pour into pit latrine, shared flush toilet, public flush toilet, or open defecation in places like rivers, gardens, sewers, ponds, cattle pens, oceans, or other unspecified locations. |
| **Liquid Waste Disposal** | Flowing sewers, non-flowing sewers/gutters, rivers. | Dumped in yard/garden, ponds, permanent holes, rivers, puddles, rice fields, oceans, or other unspecified methods. |
| **Household Waste Disposal** | Rubbish bin collected by waste management officers. | Burned, dumped into rivers, thrown in yard/garden, buried in a hole, forest, sea/lake/beach, rice fields, or other unspecified methods. |

### Mediating factor

#### Life Satisfaction

In this study, LS was measured as a mediating variable linking sanitation factors to depression. It was assessed using a single-item question from the IFLS, which asked respondents to reflect on their life as a whole and indicate their current level of satisfaction. The question was phrased: “*Reflect on your life as a whole. How satisfied are you with your life right now?*” Responses were recorded on a five-point Likert scale: 1 (Extremely satisfied), 2 (Very satisfied), 3 (Somewhat satisfied), 4 (Dissatisfied), and 5 (Very dissatisfied). For analysis, the LS variable was treated as a continuous variable.

While single-item measures may not fully capture the multidimensional nature of quality of life (QoL), this approach has been applied in several peer-reviewed studies using the same IFLS dataset for different analyses [37-39]. These studies demonstrate that the single-item LS indicator serves as a reliable and pragmatic proxy for overall subjective well-being in large-scale surveys where comprehensive QoL instruments are not available.

### Covariates

In all models, depression and life satisfaction were adjusted for age and sex, as both variables have been consistently associated with these outcomes in previous studies [40-43]. For sanitation factors, we examined the inclusion of socioeconomic factors (education level, occupation status, residency type, and wealth) due to their potential correlation with access to sanitation. However, only the model that included occupation status and residency type achieved acceptable fit indices, while those incorporating wealth and education did not converge or fell below the fit thresholds (RMSEA<0.05, CFI and TLI < 0.90). Therefore, the final models retained occupation status and residency type alongside age and sex as covariates. Including these demographic controls helps minimise confounding and ensures that the estimated direct and indirect effects of sanitation variables are adjusted for demographic differences.

## 2.3 Statistical Analysis

Descriptive statistics characterised the sample using frequencies with proportions for categorical data and mean with standard deviation (SD) for continuous variables. Pearson’s Chi-squared (χ2) test and the Kruskal-Wallis test were used to compare nominal/ordinal and continuous variables, respectively, between depressed and non-depressed groups. Structural equation modelling (SEM) was used to examine the relationships between sanitation factors and depression. SEM is a widely used analytical method in epidemiological research, particularly well-suited for analysing complex variable interactions and addressing endogeneity issues [44]. It enables the estimation of direct, indirect, and total effects of both exogenous and endogenous variables. Instead of combining all sanitation variables into a single model, we constructed and re-analysed a series of separate models for each sanitation factor. For each factor, we estimated two models: one assessing its direct association with depression and another including LS as a mediating variable. This approach enabled us to isolate the unique contribution of each sanitation factor and gain a better understanding of its relationship with both LS and depression. The pathways for each model are illustrated in Figure 1A (Model 1) and Figure 1B (Model 2). We report standardised path coefficients (β) to facilitate comparability across variables and present model fit statistics, including RMSEA, CFI, and TLI, with overall goodness-of-fit results provided in **S3 Table**. Model fit indices indicated a good overall fit across models, with SRMR values ranging from 0.004 to 0.008, RMSEA values from 0.008 to 0.020, CFI values from 0.972 to 0.994, and TLI values from 0.897 to 0.987. Before performing the SEM analysis, a correlation matrix was examined to assess bivariate associations among sanitation variables, life satisfaction, and depression (**S2 Table**).

Figure 1. SEM pathways: (A) Direct effect of sanitation on depression; (B) Indirect effect via life satisfaction.

# 3. Results

## 3.1 Sample characteristics and bivariate analysis

Table 2 presents the descriptive characteristics stratified by depression status. In total, 53% of the sample consisted of women, with an average age of 37.32 years (±14.93 years) among all participants. Most of the sample resides in urban areas (59%), has completed senior high school (55%), and employed (67%). Regarding access to basic amenities, the majority reported using safe drinking water (98%), improved water sources (96%), improved toilet facilities (74%), and safe liquid waste disposal (67%). However, only 35% of the sample have access to improved waste disposal. Details on combinations of water and sanitation issues, as well as the cumulative number of issues reported by participants, are provided in supplementary S4 and S5 Tables.

The bivariate analysis (last column of Table 2) reveals that individuals with depression are less likely to have access to safe drinking water (p ≤ 0.001), improved water sources (p = 0.023), improved toilet facilities (p ≤ 0.001), and improved waste disposal methods (p = 0.004). Furthermore, there is a significant gender disparity in depression rates, with a higher proportion of females affected compared to males (p=0.005). Age also plays a crucial role, as younger individuals are more prone to depression (p≤0.001). Additionally, individuals with depression are more likely to have lower educational attainment, with 54% having completed senior high school compared to only 13% being college graduates. Economic disparities are evident, with those in lower economic quintiles more likely to suffer from depression (20% in quintile 1 vs 18% in quintile 5). Lastly, the prevalence of depression was substantially higher among those who reported being dissatisfied or very dissatisfied with life, supporting the notion that depression and life satisfaction follow an inverse and potentially U-shaped relationship.

**Table 2.** **Descriptive statistics on the individual characteristics.**

| **Variables** | **Total  (n = 31,446)** | | **No depression**  **(n= 24,134)** | | | | **Depression**  **(n= 7,312)** | | | | **P value*** | | **Missing data** | |
| --- | --- | --- | --- | --- | --- | --- | --- | --- | --- | --- | --- | --- | --- | --- |
|  | **N** | **%/SD** | **n /mean** | | **% /SD** | | **n/mean** | | **%/SD** | |  |  | **N (%)** | |
| **Safe drinking water** | 31,023 | 98.65 | 23,845 | 98.80 | | 7,178 | | 98.17 | | <0.001 | | 44 (0.13) | |  |
| **Improved water source** | 27,149 | 86.34 | 20,974 | 86.91 | | 6,175 | | 84.45 | | <0.001 | | 44 (0.13) | |  |
| **Improved toilet facility** | 23,447 | 74.56 | 18,235 | 75.56 | | 5,212 | | 71.28 | | <0.001 | | - | |  |
| **Safe liquid waste disposal** | 21,006 | 66,89 | 16,128 | 66.83 | | 4,878 | | 66.71 | | 0.950 | | 44 (0.13) | |  |
| **Safe waste disposal** | 11,114 | 35.34 | 8,633 | 35.77 | | 2,481 | | 33.93 | | 0.004 | | - | |  |
| **Urban residence** | 18,558 | 59,02 | 14,207 | 58.87 | | 4,351 | | 59.50 | | 0.331 | | - | |  |
| **Age (years), mean (SD)** | 37.32 | 14.93 | 38.16 | 15.03 | | 34.56 | | 14.24 | | <0.001 | | 8 (0.02) | |  |
| **Sex** |  |  |  |  | |  | |  | | 0.005 | | - | |  |
| **Male** | 14,700 | 46.76 | 11,403 | 47.25 | | 3,297 | | 45.09 | |  | |  | |  |
| **Female** | 16,738 | 53.24 | 12,725 | 52.73 | | 4,013 | | 54.88 | |  | |  | |  |
| **Education** |  |  |  |  | |  | |  | | <0.001 | | 1,222 (3.88) | |  |
| **Primary school and lower** | 9,291 | 30.74 | 7,099 | 29.41 | | 2,192 | | 29.98 | |  | |  |  |  |
| **Senior high school and higher** | 16.578 | 54.85 | 12,612 | 52.26 | | 3,966 | | 54.24 | |  | |  |  |  |
| **College** | 43.55 | 14,41 | 3,433 | 14.22 | | 922 | | 12.61 | |  | |  |  |  |
| **Occupation status** |  |  |  |  | |  | |  | | 0.055 | | 2 (0.01) | |  |
| **Employed** | 21,031 | 66,88 | 16,220 | 67.21 | | 4,811 | | 65.80 | |  | |  | |  |
| **Unemployed** | 10,413 | 33,12 | 7,913 | 32.79 | | 2,500 | | 34.19 | |  | |  | |  |
| **Economic status** |  |  |  |  | |  | |  | | <0.001 | | 2,071 (6.58) | |  |
| **Quintile 5** | 5,910 | 20.12 | 4,608 | 19.09 | | 1,302 | | 17.81 | |  | |  |  |  |
| **Quintile 4** | 5,931 | 20.19 | 4,600 | 19.06 | | 1,331 | | 18.20 | |  | |  |  |  |
| **Quintile 3** | 5,911 | 20.12 | 4,503 | 18.66 | | 1,408 | | 19.26 | |  | |  |  |  |
| **Quintile 2** | 5,863 | 19.96 | 4,502 | 18.65 | | 1,361 | | 18.61 | |  | |  |  |  |
| **Quintile 1** | 5,760 | 19.61 | 4,300 | 17.82 | | 1,460 | | 19.97 | |  | |  |  |  |
| **Life satisfaction** |  |  |  |  | |  | |  | | <0.001 | | 4 (0.01) | |  |
| **Extremely satisfied** | 1,340 | 4.26 | 1,043 | 4.32 | | 297 | | 4.06 | |  | |  | |  |
| **Very satisfied** | 12,364 | 39.32 | 10,026 | 41.54 | | 2,338 | | 31.97 | |  | |  | |  |
| **Somewhat satisfied** | 13,346 | 42.44 | 10,235 | 42.41 | | 3,111 | | 42.55 | |  | |  | |  |
| **Dissatisfied** | 3,889 | 12.37 | 2,558 | 10.60 | | 1,331 | | 18.20 | |  | |  | |  |
| **Very dissatisfied** | 504 | 1.60 | 271 | 1.12 | | 233 | | 3.19 | |  | |  | |  |

**Note:** * Bivariate analysis was performed using Chi-square for categorical variables and Kruskal-Wallis for continuous variables.

## 3.2 SEM Analysis

Detailed results from the SEM analysis, including direct and indirect effects, are presented in Table 3. In the first model, we found that drinking water (β=0.022, p<0.001), water source (β=0.033, p<0.001), toilet facilities (β=0.039, p<0.001), and waste disposal method (β=0.021, p<0.001) were significantly associated with depression. In contrast, the method of sewage disposal was not significantly related to depression. These findings suggest that inadequate access to safe water, improved sanitation access, and appropriate waste management are important predictors of depressive symptoms.

In the second SEM model (Figure 1B), significant direct effects of unimproved sanitation on depression were observed for drinking water (β=0.021, p<0.001), water source (β=0.026, p<0.001), toilet facilities (β=0.031, p<0.001), and waste disposal method (β=0.015, p<0.05). These results are consistent with the direct pathways identified in Model A, reinforcing that these four sanitation factors exert a direct influence on depression even when life satisfaction is included as a mediator. Similar to Model A, sewage disposal method was not significantly associated with depression, nor did it show an indirect effect through life satisfaction.

Regarding the relationship between sanitation factors and LS, unimproved sanitation was also significantly linked to lower LS, especially for drinking water (β=0.013, p<0.001), water source (β=0.054, p<0.001), toilet facilities (β=0.068, p<0.001), and waste disposal methods (β=0.046, p<0.001), suggesting a possible indirect pathway. Finally, LS was strongly associated with depression across all sanitation factors (β ≈ 0.124–0.126, p<0.001), confirming its mediating role in the sanitation–depression relationship. These results suggest that the effect of unimproved sanitation on depression is partly mediated through reduced LS, though substantial direct effects remain for several sanitation dimensions. Supplementary analyses, including logistic regression models (**S6 Table**), supported these findings with consistent associations, and a sub-sample SEM for the lowest wealth quintile (**S7 Table**) showed similar patterns. Some effects became non-significant, likely due to a smaller sample size and less variability in the lower-income group.

**Table 3. Standardised Coefficients for Direct Effects of Sanitation on Depression (Model A) and Indirect Effects via LS as a Mediator (Model B), SEM Analysis**

| **Sanitation Variables** | **Direct – Model A** | **Indirect – Model B** | | |
| --- | --- | --- | --- | --- |
|  | **Depression** | **Sanitation 🡪 Depression** | **Sanitation 🡪 LS** | **LS 🡪 Depression** |
| **Drinking water** | 0.022 (0.011 – 0.033)^*^ | 0.021 (0.010 – 0.032)^*^ | 0.013 (0.001 – 0.023)^+^ | 0.125 (0.114 – 0.136)^*^ |
| **Water source** | 0.033 (0.022 – 0.444)^*^ | 0.026 (0.015 – 0.037)^*^ | 0.054 (0.043 – 0.065)^*^ | 0.124 (0.113 – 0.135)^*^ |
| **Toilet facilities** | 0.039 (0.028 – 0.050)^*^ | 0.031 (0.020 – 0.042)^*^ | 0.068 (0.057 – 0.079)^*^ | 0.124 (0.113 – 0.135)^*^ |
| **Sewage disposal method** | 0.004 (-0.006 – 0.015) | 0.003 (-0.007 – 0.014) | 0.010 (-0.001 – 0.021) | 0.126 (0.115 – 0.136)^*^ |
| **Waste disposal method** | 0.021 (0.010 – 0.032)^*^ | 0.015 (0.004 – 0.026)^+^ | 0.046 (0.035 – 0.057)^*^ | 0.125 (0.114 – 0.136)^*^ |

Note: Standardized coefficient; ^+^p<0.05, ^#^<0.005, ^*^p<0.001. Sanitation factors were adjusted with employment status and residency type, while LS and depression were adjusted with age and sex.

# 4. Discussion

This study is the first to investigate the relationship between sanitation and depression in Indonesia, employing SEM analysis with life satisfaction as a mediator. The findings contribute new insights into the pathways linking sanitation, life satisfaction, and mental health. The following discussion integrates these results with recent empirical literature, including reviewer-suggested studies, and highlights implications for theory, policy, and future research.

## 4.1 Sanitation and depression

The SEM analysis indicates that unsafe drinking water, unimproved water sources, unimproved toilets, and unsafe waste disposal were directly associated with higher odds for depression. Several sanitation factors were also associated with lower LS, which in turn was strongly linked to depression, confirming its role as a mediator in the sanitation–mental health pathway. This study strengthens the existing literature that links poor sanitation conditions to increased levels of depression [20, 23-25, 35, 45-49], highlighting the importance of improving sanitation to enhance mental health outcomes.

We identified a significant association between unimproved drinking water quality and depression, consistent with studies which demonstrated that consumption of water contaminated with heavy metals such as copper, cadmium [24], lead, nitrate, and arsenic [20] was associated with higher rates of depression. Reflecting similar findings in Indonesia, a study showed that relying on unimproved water sources and drinking unboiled water considerably raised the risk of depression [35]. Beyond drinking water quality, we also found that the use of unimproved water sources for daily activities was significantly associated with higher odds of depression. This finding aligns with studies from Canada [21] and South Africa [47], which reported that individuals with piped household water sources had lower odds of depression. Further evidence from the China Family Panel Studies showed that access to clean water was associated with lower levels of depression [45].

Unsafe drinking water can affect mental health through multiple interconnected pathways. First, unsafe water compromises physical health by increasing the risk of diarrhoea, acute respiratory infections, and undernutrition [50], which may indirectly contribute to depression through reduced energy, chronic illness, and feelings of hopelessness. Second, inadequate or unsafe water access acts as a persistent external stressor, generating fear of disease, household conflict, and even trauma associated with water-related illness [51]. Beyond physical health, limited access to safe water and adequate sanitation can heighten psychosocial stress, including worry, shame, and reduced privacy. For women in particular, water insecurity has been linked to increased fear of harassment or physical violence when collecting water, which exacerbates anxiety and psychological distress [14].

Furthermore, this study identified a significant direct association between the use of unimproved toilet facilities and depression, which aligns with studies conducted globally. A WHO SAGE wave 2 study reported that individuals using unimproved toilet facilities were more likely to report major depressive episodes [23]. Additionally, a study in China indicated that deficiencies in village infrastructure, including inadequate drinking water and toilet facilities, were positively associated with higher depression rates [46]. Studies from Kenya, Bangladesh, and Dhaka further support these findings, showing that regular access to better-quality toilets and latrines has a significant positive impact on mental health and well-being [11, 13, 25].

A plausible mechanism linking unimproved sanitation to poor mental well-being is outlined in Sclar’s conceptual model [52]. Lack of privacy and safety during open defecation or using inadequate facilities can cause anxiety, shame, and loss of dignity. This issue is even more severe among women and girls who face risks during urination, defecation, and menstruation [20]. Fear and anxiety often accompany perceived risks to safety, while actual violations, such as poorly maintained facilities or the need to practice open defecation, exacerbate feelings of shame and psychological stress, ultimately contributing to depression [14]. These experiences are further shaped by factors such as gender identity, physical ability, life stage, residency status, and socioeconomic status [52].

Further analysis revealed a significant pathway between the use of unimproved waste disposal methods and depression. Methods such as burning waste, dumping it into rivers, disposing of it in gardens, or burying it in holes can lead to significant pollution near residential areas. A study from South Africa discovered that residing within 5 km of a waste site was significantly associated with depression [49]. Similarly, a Dutch study found that higher levels of odour annoyance from sources such as sewerage and others were linked to increased psychological distress and a higher likelihood of clinical depression [48]. Improper waste disposal can contribute to depression through multiple pathways. Open burning of mixed household waste releases harmful air pollutants, such as particulate matter (PM) [53]. Exposure to such air pollutants was known to be significantly associated with increased severity of depression among asthma patients [54] and even higher risk of suicide [55]. Beyond air pollutants, waste left in open dumps releases toxic gases and foul odours, which may heighten psychological stress, reduce LS, and contribute to emotional distress, thereby increasing vulnerability to depression [56, 57].

## 4.2 Life satisfaction as a mediator

The current study showed that life satisfaction partially mediates the relationship between sanitation factors and depression. Specifically, better sanitation was associated with higher LS, which in turn was linked to lower depressive symptoms. Our finding that sanitation is linked to LS aligns with other LMIC studies [58-60]. For example, in Sucre, Bolivia, improved water access, better quality, fewer interruptions, and easier access were strongly associated with higher LS [58]. Similarly, an analysis in Indonesia showed households with private improved latrines had the highest life satisfaction, while those sharing facilities or practising open defecation reported lower LS [59]. Complementing these findings, a Chilean study revealed that residential air pollution was strongly and consistently negatively associated with LS, particularly in areas where non-emitting households are exposed to pollution generated by others [60].

Plausible mechanisms help explain how sanitation influences LS. Improved sanitation not only enhances physical conditions but also contributes to psychological and social well-being [61]. Access to clean and private facilities enhances comfort and self-esteem, while also reducing psychological distress. These improvements can indirectly boost LS by promoting better health, reducing healthcare costs, and saving time that would otherwise be spent coping with inadequate facilities [61]. Additionally, a qualitative study highlights that sanitation shapes LS by creating a sense of safety and control over one’s environment, alleviating shame and stress, and ensuring privacy, all of which foster a more secure, dignified, and emotionally supportive living context [10].

In this study, we found that LS was strongly associated with depression across all sanitation factors, consistent with evidence from other settings. In Kampala, Uganda, lower LS were associated with higher stress levels and frequent depressive symptoms [62]. Similarly, a study in Korea [63] and Portugal [64] found that participants with higher LS had a lower risk of depression. These findings are supported by research suggesting plausible mechanisms through which LS influences depression: higher LS appears to protect against depressive symptoms by enhancing resilience, positive coping, and social support, while reducing vulnerability to negative emotional states [65, 66]. LS may also indirectly influence depression by supporting daily functioning and overall well-being [67].

## 4.3 Theoretical and practical implications

The mediation model examined in this study aligns with recent empirical frameworks highlighting the role of sanitation factors in shaping mental health outcomes [51, 52, 55]. While prior research has often focused on the direct impact of sanitation on physical health or depression in isolation, more integrated approaches have demonstrated that factors such as life satisfaction can serve as mediators in these pathways. For example, improvements in sanitation have been shown to decrease the odds of depression [21, 25, 45, 48], with factors such as LS moderating this pathway [62-64].

Despite growing recognition of the environmental determinants of mental health, limited empirical work has examined the role of material well-being, particularly water and sanitation conditions, using advanced analytical approaches such as structural equation modelling to elucidate their pathways to mental health outcomes [14, 52]. This study contributes to this gap by applying a moderated mediation model to explore both the direct and indirect associations between multiple sanitation dimensions and depression, providing a more nuanced understanding of how environmental living conditions influence psychological well-being.

From a practical perspective, expanding access to safe drinking water and improved sanitation is a practical and cost-effective way to enhance the well-being of populations. Evidence from Li et al. indicates that better village infrastructure and community resources are linked to fewer depressive symptoms, highlighting the mental health benefits of such investments [46]. WHO further estimated that every US$1 invested in sanitation yields a return of US$5.50 through reduced healthcare costs, higher productivity, and fewer premature deaths [8]. Integrating water and sanitation programs into national mental health and development agendas could therefore generate both health and economic gains, particularly in underserved rural settings.

## 4.4 Strengths, limitations, and directions for future research

The strength of this study lies in its use of a large, nationally representative sample from Indonesia, enhancing the generalizability of its findings to similar LMIC contexts. By examining a range of sanitation factors individually, this study provides a detailed assessment of their direct associations with depression as well as their indirect effects mediated through life satisfaction. The use of SEM further enables the simultaneous estimation of these pathways, providing insights into the mechanisms that link environmental conditions to mental health outcomes.

However, this study also has notable limitations. The data were collected in 2014–2015, and changes in sanitation infrastructure and mental health awareness since then may affect the current relevance of the findings. Additionally, the cross-sectional nature of the data limits the ability to make causal inferences. Third, the IFLS only covers 83% of the population and excludes eastern regions of Indonesia, where sanitation conditions are likely to be more challenging. Missing data, although relatively small (0.01 - 6.58%), were not systematically addressed. These limitations may affect the comprehensiveness and accuracy of the findings, potentially skew the results and limit the generalizability of the study’s conclusions.

Future research should build on these findings by using more recent and longitudinal data to capture changes in sanitation infrastructure and mental health trends, enabling stronger causal inference. Expanding coverage to eastern regions of Indonesia and other underrepresented populations would enhance generalizability. Additionally, exploring other determinants of depression, such as social support, economic status, and environmental stressors, could provide a more comprehensive understanding of how sanitation affects mental health.

# 5. Conclusion

Our study highlights the multifaceted relationship between water, sanitation, and mental health in Indonesia, revealing that inadequate access to safe drinking water, improved sanitation facilities, and proper waste management are significant predictors of depressive symptoms. Even after accounting for life satisfaction as a mediator, these environmental factors maintained strong direct effects on depression, underscoring their independent influence. The results also demonstrate that unimproved sanitation contributes to lower life satisfaction, which in turn exacerbates depression, suggesting that the mental health burden linked to poor sanitation operates through both direct and psychosocial pathways. Our research suggests that expanding access to safe drinking water and sanitation is a cost-effective way to improve population health, with evidence showing mental health benefits and high economic returns on investment. Integrating these initiatives into national policies, especially in underserved rural areas, could lead to substantial health and economic gains.

# Acknowledgements

This research was based on the IFLS-5 conducted by RAND (http://www.rand.org/labor/FLS/IFLS.html). The authors extend their gratitude to RAND for providing access to the survey data and to the study participants for their valuable contributions.

# Competing Interests

The authors declare no competing interests.

# Ethics Statement

The IFLS has been approved by ethics review boards at both RAND Corporation and Gadjah Mada University in Indonesia, with written informed consent obtained from all respondents before data collection. As the study utilised publicly available IFLS datasets containing no personal identification, no further ethical approval was required. The confidentiality and anonymity of participants’ names, addresses, locations, and neighbourhoods were maintained by the data source, in accordance with RAND's human subjects’ protection rules. Respondents were assured that their answers would remain confidential and their identities would only be linked to an anonymous code. The use of the data set for this study has also been approved by the University of Manchester, the Faculty of Nursing Universitas Indonesia, and the National Research and Innovation Agency of Indonesia (BRIN).

# Funding

This research was funded by the NIHR [Global Health Research for Sustainable Care for anxiety and depression in Indonesia (Award ID NIHR134638)] using UK international development funding from the UK Government to support global health research. The views expressed in this publication are those of the author(s) and not necessarily those of the NIHR or the UK government.

# Author contributions

- Conceptualisation and methodology: Mashita Fajri, Asri Maharani
- Data cleaning: Mashita Fajri
- Formal analysis: Mashita Fajri, Asri Maharani
- Data interpretation: Mashita Fajri, Asri Maharani, Jonathan Gibson, Sri Idaiani
- Supervision: Sri Idaiani, Herni Susanti
- Writing – original draft: Mashita Fajri, Asri Maharani
- Writing – review & editing: Amy Blakemore, Jonathan Gibson, Sri Idaiani, Penny Bee, Helen Brooks, Herni Susanti
- Funding acquisition: Penny Bee, Helen Brooks, Herni Susanti

# Supporting information

**S1 Table. Complete Sanitation and Water Safety Classification Table.**

**S2 Table. Correlation Matrix**

**S3 Table. Overall goodness-of-fit statistics of each model**

**S4 Table. Combinations of Water and Sanitation Issues**

**S5 Table. Cumulative Sanitation Issues and Depression**

**S6 Table. Robustness Check Using Logistic Regression Models**

**S7 Table. Structural Equation Model Results Among the Poorest 20th Percentile**

# References

1.	GBD Mental Disorders Collaborators. Global, regional, and national burden of 12 mental disorders in 204 countries and territories, 1990–2019: a systematic analysis for the Global Burden of Disease Study 2019. The Lancet Psychiatry. 2022;9(2):137-50. doi: doi:10.1016/S2215-0366(21)00395-3.

2.	WHO. Depressive disorder (depression): World Health Organization; 2024 [updated 31 March 2023]. Available from: <https://www.who.int/news-room/fact-sheets/detail/depression> [accessed 9 Sept 2024]

3.	Dybdahl R, Lien L. Mental health is an integral part of the sustainable development goals. Preventive Medicine and Community Health. 2018;1. doi: doi:10.15761/PMCH.1000104.

4.	WHO. Depression and Other Common Mental Disorders: Global Health Estimates Geneva: World Health Organization; 2017. Available from: <https://www.who.int/publications/i/item/depression-global-health-estimates> [accessed 20 June 2024].

5.	Arizal M, Wisana IDGK. Mental Health Effects on Job Retention in Indonesia. Journal of Developing Economies. 2023;8(1):1-20. doi: 10.20473/jde.v8i1.37445.

6.	Setiyawati D, Puspakesuma N, Jatmika W, Colucci E. Indonesian Stakeholders' Perspectives on Warning Signs and Beliefs about Suicide. Behavioral sciences (Basel, Switzerland). 2024;14. doi: 10.3390/bs14040295.

7.	Puspitasari IM, Sinuraya RK, Rahayu C, Witriani W, Zannah U, Hafifah A, et al. Medication Profile and Treatment Cost Estimation Among Outpatients with Schizophrenia, Bipolar Disorder, Depression, and Anxiety Disorders in Indonesia. Neuropsychiatric Disease and Treatment. 2020;16:815-28. doi: doi:10.2147/NDT.S240058.

8.	WHO. Sanitation: World Health Organization; 2024 [updated 22 March 20249 September 2024]. Available from: <https://www.who.int/news-room/fact-sheets/detail/sanitation> [Accessed: 9 October 2025].

9.	UNICEF, WHO. State of the World’s Sanitation New York: UNICEF and WHO; 2020 Available from: <https://www.unicef.org/media/86836/file/State-of-the-world%E2%80%99s-sanitation-2020.pdf> [Accessed: 9 September 2024].

10.	Ross I, Cumming O, Dreibelbis R, Adriano Z, Nala R, Greco G. How does sanitation influence people's quality of life? Qualitative research in low-income areas of Maputo, Mozambique. Social Science & Medicine. 2021;272:113709. doi: [10.1016/j.socscimed.2021.113709](https://doi.org/10.1016/j.socscimed.2021.113709).

11.	Hoque F, Khan MA, Preya I. Implications of sanitation environment on women's health: a case on railway slum of Khulna city in Bangladesh. Journal of Science Technology and Environment Informatics. 2022;12:775-85. doi: 10.18801/jstei.120122.78.

12.	Caruso BA, Cooper HLF, Haardörfer R, Yount KM, Routray P, Torondel B, et al. The association between women's sanitation experiences and mental health: A cross-sectional study in Rural, Odisha India. SSM Popul Health. 2018;5:257-66. doi: 10.1016/j.ssmph.2018.06.005.

13.	Winter SC, Obara LM, Barchi F. Environmental Correlates of Health-Related Quality of Life among Women Living in Informal Settlements in Kenya. Int J Environ Res Public Health. 2019;16(20). doi: 10.3390/ijerph16203948.

14.	Kimutai JJ, Lund C, Moturi WN, Shewangizaw S, Feyasa M, Hanlon C. Evidence on the links between water insecurity, inadequate sanitation and mental health: A systematic review and meta-analysis. PLoS One. 2023;18(5):e0286146. doi: 10.1371/journal.pone.0286146.

15.	Filipcic I, Simunovic Filipcic I, Ivezic E, Matic K, Tunjic Vukadinovic N, Vuk Pisk S, et al. Chronic physical illnesses in patients with schizophrenia spectrum disorders are independently associated with higher rates of psychiatric rehospitalization; a cross-sectional study in Croatia. Eur Psychiatry. 2017;43:73-80. doi: 10.1016/j.eurpsy.2017.02.484.

16.	Spurrier GF, Shulman K, Dibich S, Benoit L, Duckworth K, Martin A. Physical symptoms as psychiatric manifestations in medical spaces: A qualitative study. Front Psychiatry. 2022;13:1074424. doi: 10.3389/fpsyt.2022.1074424.

17.	Kalonia N, Garhwal K, Singh V. Life Satisfaction and Resilience as a Predictor of Psychological Well-being among Youth. The International Journal of Indian Psychology. 2023;10:1004-10. doi: 10.25215/1004.098.

18.	Qamar S, Yaqoob N. Positivity level predicts better health and mental health among adults. Journal of Current Health Sciences. 2023;3(1):13-20. doi: 10.47679/jchs.202345.

19.	Gan DRY, Wister AV, Best JR. Environmental Influences on Life Satisfaction and Depressive Symptoms Among Older Adults With Multimorbidity: Path Analysis Through Loneliness in the Canadian Longitudinal Study on Aging. Gerontologist. 2022;62(6):855-64. doi: 10.1093/geront/gnac004.

20.	Manczak EM, Miller JG, Gotlib IH. Water contaminant levels interact with parenting environment to predict development of depressive symptoms in adolescents. Dev Sci. 2020;23(1):e12838. doi: 10.1111/desc.12838.

21.	O'Gorman M. Mental and physical health impacts of water/sanitation infrastructure in First Nations communities in Canada: An analysis of the Regional Health Survey. World Development. 2021;145:105517. doi: [10.1016/j.worlddev.2021.105517](https://doi.org/10.1016/j.worlddev.2021.105517).

22.	Blay SL, Schulz AJ, Mentz G. The Relationship of Built Environment to Health-Related Behaviors and Health Outcomes in Elderly Community Residents in a Middle Income Country. J Public Health Res. 2015;4(2):548. doi: 10.4081/jphr.2015.548.

23.	Simiyu S, Bagayoko M, Gyasi RM. Associations between water, sanitation, and depression among older people in Ghana: empirical evidence from WHO-SAGE Wave 2 survey. Aging & Mental Health. 2022;26(6):1112-9. doi: 10.1080/13607863.2021.1910796.

24.	Zhou S, Su M, Shen P, Yang Z, Chai P, Sun S, et al. Association between drinking water quality and mental health and the modifying role of diet: a prospective cohort study. BMC Medicine. 2024;22(1):53. doi: 10.1186/s12916-024-03269-3.

25.	Gruebner O, Khan MM, Lautenbach S, Müller D, Krämer A, Lakes T, et al. Mental health in the slums of Dhaka - a geoepidemiological study. BMC Public Health. 2012;12:177. doi: 10.1186/1471-2458-12-177.

26.	Afifah T, Nuryetty MT, Cahyorini, Musadad DA, Schlotheuber A, Bergen N, et al. Subnational regional inequality in access to improved drinking water and sanitation in Indonesia: results from the 2015 Indonesian National Socioeconomic Survey (SUSENAS). Glob Health Action. 2018;11(sup1):1496972. doi: 10.1080/16549716.2018.1496972.

27.	Sai A, Al Furqan R, Ushijima K, Hamidah U, Ikemi M, Rani W, et al. Physical and Mental Health of Sanitation Workers in an Urban Slum of Indonesia: Personal Hygiene and the Construction of Self-Esteem in Waste-Handling2021.

28.	TW Sukesi, S Sulistyawati, U Khair, SA Mulasari, F Tentama, FA Ghazali, et al. Hubungan antara Kesehatan Lingkungan dengan Gangguan Emosional. Jurnal Kesehatan Lingkungan Indonesia. 2023;22(2). doi: 10.14710/jkli.22.2.128-133.

29.	Dwipayanti NMU, Rutherford S, Phung D, Chu C. How Important is Culture to Sanitation Uptake? The Influence of Local Values in Rural Bali. Advanced Science Letters as Conference Proceeding of International Conference on Public Health for Tropical and Coastal Development (ICOPH-TCD 2016). 2017;23. doi: 10.1166/asl.2017.9160.

30.	Strauss J, Witoelar F, Sikoki B. The Fifth Wave of the Indonesia Family Life Survey: Overview and Field Report. RAND Corporation, 2016.

31.	RAND. RAND IFLS-5 Survey Description 2015 [12 July 2024]. Available from: <https://www.rand.org/labor/FLS/IFLS.html>

32.	Andresen EM, Malmgren JA, Carter WB, Patrick DL. Screening for depression in well older adults: evaluation of a short form of the CES-D (Center for Epidemiologic Studies Depression Scale). Am J Prev Med. 1994;10(2):77-84. PubMed PMID: 8037935.

33.	James C, Powell M, Seixas A, Bateman A, Pengpid S, Peltzer K. Exploring the psychometric properties of the CES-D-10 and its practicality in detecting depressive symptomatology in 27 low- and middle-income countries. Int J Psychol. 2020;55(3):435-45. doi: 10.1002/ijop.12613.

34.	Fahmi M, Panjaitan NA, Habibie I, Siregar AYM, Amarullah G, Rahma, et al. Does your neighborhood protect you from being depressed? A study on social trust and depression in Indonesia. BMC Public Health. 2019;19(1):1371. doi: doi:10.1186/s12889-019-7657-5.

35.	Hilal NA-DM, Al-Aghbari N. Water and sanitation quality on depressive symptoms across urban and rural areas in Indonesia: evidence from IFLS-5 data. International Journal of Environmental Health Research.1-12. doi: 10.1080/09603123.2025.2498063.

36.	Islam MA, Rahman M, Uddin MF, Tariqujjaman M, Karmakar G, Rahman MA, et al. Household food insecurity and unimproved toilet facilities associate with child morbidity: evidence from a cross-sectional study in Bangladesh. BMC Public Health. 2022;22(1):1075. doi: 10.1186/s12889-022-13469-2.

37.	Renanita T, Rembulan CL. The Effects of Social Comparison and Subjective Financial Well-Being on Subjective Well-Being. Europe’s Journal of Psychology. 2025;21(3):179-93. doi: 10.5964/ejop.14791.

38.	Choirina V, Ayriza Y, Wibowo YS. Religiosity and Life Satisfaction in Indonesia: Evidence from a Community Survey. Journal of Educational, Health and Community Psychology. 2021;10:38. doi: 10.12928/jehcp.v10i1.19625.

39.	Pratiwi LLE, Asri Y, Patria DKA, Nurmayunita H. *Hubungan Kepuasan Hidup (Life Satisfaction) dengan Terjadinya Depresi pada Lansa di Indonesia: Studi Indonesia Family Life Survey Wave-5 (IFLS-5). Jurnal Ilmu Kesehatan*. 2025;(2):160-9%V 13. doi: 10.32831/jik.v13i2.783. [Life Satisfaction and the Occurrence of Depression among Elderly in Indonesia: Indonesian Family Life Survey Wave-5 (IFLS-5). Journal of Health Sciences.]

40.	Sikström S, Kelmendi B, Persson N. Assessment of depression and anxiety in young and old with a question-based computational language approach. npj Mental Health Research. 2023;2(1):11. doi: 10.1038/s44184-023-00032-z.

41.	Park HY. Sex/Gender Differences in Depression and Anxiety Disorders. In: Kim N, editor. Sex/Gender-Specific Medicine in Clinical Areas. Singapore: Springer Nature Singapore; 2024. p. 369-79.

42.	Joshanloo M, Jovanović V. The relationship between gender and life satisfaction: analysis across demographic groups and global regions. Arch Womens Ment Health. 2020;23(3):331-8. doi: 10.1007/s00737-019-00998-w.

43.	Kaiser M, Otterbach S, Sousa-Poza A. Using machine learning to uncover the relation between age and life satisfaction. Scientific Reports. 2022;12(1). doi: 10.1038/s41598-022-09018-x.

44.	Amorim LD, Fiaccone RL, Santos CA, Santos TN, Moraes LT, Oliveira NF, et al. Structural equation modeling in epidemiology. Cad Saude Publica. 2010;26(12):2251-62. doi: 10.1590/s0102-311x2010001200004.

45.	Shi H. Depression: Does water matter? Water Policy. 2022;24. doi: 10.2166/wp.2022.263.

46.	Li LW, Liu J, Zhang Z, Xu H. Late-life depression in Rural China: do village infrastructure and availability of community resources matter? Int J Geriatr Psychiatry. 2015;30(7):729-36. doi: 10.1002/gps.4217.

47.	Mngadi LC, Cuadros DF, Tanser F, Burns JK, Slotow R, Tomita A. Water, sanitation and depression in rural communities: evidence from nationally representative study data in South Africa. Psychology, Health & Medicine. 2023;28(9):2441-9. doi: 10.1080/13548506.2023.2179643.

48.	Berkers E, Pop I, Cloïn M, Eugster A, van Oers H. The relative effects of self-reported noise and odour annoyance on psychological distress: Different effects across sociodemographic groups? PLoS One. 2021;16(10). doi: 10.1371/journal.pone.0258102.

49.	Tomita A, Cuadros DF, Burns JK, Tanser F, Slotow R. Exposure to waste sites and their impact on health: a panel and geospatial analysis of nationally representative data from South Africa, 2008 & 2013;2015. The Lancet Planetary Health. 2020;4(6):e223-e34. doi: 10.1016/S2542-5196(20)30101-7.

50.	Wolf J, Johnston RB, Ambelu A, Arnold BF, Bain R, Brauer M, et al. Burden of disease attributable to unsafe drinking water, sanitation, and hygiene in domestic settings: a global analysis for selected adverse health outcomes. The Lancet. 2023;401(10393):2060-71. doi: 10.1016/S0140-6736(23)00458-0.

51.	Toivettula A, Varis O, Vahala R, Juvakoski A. Making waves: Mental health impacts of inadequate drinking water services — From sidenote to research focus. Water Research. 2023;243. doi: [10.1016/j.watres.2023.120335](https://doi.org/10.1016/j.watres.2023.120335).

52.	Sclar GD, Penakalapati G, Caruso BA, Rehfuess EA, Garn JV, Alexander KT, et al. Exploring the relationship between sanitation and mental and social well-being: A systematic review and qualitative synthesis. Soc Sci Med. 2018;217:121-34. doi: 10.1016/j.socscimed.2018.09.016.

53.	Mihai FC, Banica A, Grozavu A. Backyard burning of household waste in rural areas. Environmental impact with focus on air pollution. 19th International Multidisciplinary Scientific GeoConference on Ecology, Economics, Education and Legislation; Albena, Bulgaria: HAL Open Science; 2019. p. 55-62.

54.	Maheshwari V, Negandhi P, Jaggi V, Batra S, Prabhakaran P, Menon JS. Association of Short-Term Exposure to Air Pollution with Depression in Patients with Asthma: A Cross-Sectional Study in Delhi, India. Journal of Health and Pollution. 2024;12(1-4). doi: doi:10.1289/JHP1003.

55.	Liu Q, Wang W, Gu X, Deng F, Wang X, Lin H, et al. Association between particulate matter air pollution and risk of depression and suicide: a systematic review and meta-analysis. Environ Sci Pollut Res Int. 2021;28(8):9029-49. doi: 10.1007/s11356-021-12357-3.

56.	Marin C, Alobid I, Fuentes M, López-Chacón M, Mullol J. Olfactory Dysfunction in Mental Illness. Current Allergy and Asthma Reports. 2023;23(3):153-64. doi: 10.1007/s11882-023-01068-z.

57.	Siddiqua A, Hahladakis JN, Al-Attiya WAKA. An overview of the environmental pollution and health effects associated with waste landfilling and open dumping. Environmental Science and Pollution Research. 2022;29(39). doi: 10.1007/s11356-022-21578-z.

58.	Guardiola J, García-Rubio MA, Guidi-Gutiérrez E. Water Access and Subjective Well-Being: The Case of Sucre, Bolivia. Applied Research in Quality of Life. 2014;9(2):367-85. doi: 10.1007/s11482-013-9218-x.

59.	Nelson KB, Karver J, Kullman C, Graham JP. User Perceptions of Shared Sanitation among Rural Households in Indonesia and Bangladesh. PLOS ONE. 2014;9(8). doi: 10.1371/journal.pone.0103886.

60.	Vienne Arancibia V. Does it matter where it comes from? Urban air pollution and life satisfaction. Journal of Environmental Management. 2025;387:125602. doi: [10.1016/j.jenvman.2025.125602](https://doi.org/10.1016/j.jenvman.2025.125602).

61.	Mohetaer Y, Han C, Meng L, Shangguan Y, Liu P. From Flush to Fulfillment: Unveiling the Influence of Upgraded Sanitation on Subjective Well-Being. Social Indicators Research. 2025;178:335-70. doi: 10.1007/s11205-025-03589-8.

62.	Culbreth RE, Nielsen KE, Mobley K, Palmier J, Bukuluki P, Swahn MH. Life Satisfaction Factors, Stress, and Depressive Symptoms among Young Women Living in Urban Kampala: Findings from the TOPOWA Project Pilot Studies. International Journal of Environmental Research and Public Health. 2024;21(2):184. doi:10.3390/ijerph21020184.

63.	Seo EH, Kim S-G, Kim SH, Kim JH, Park JH, Yoon H-J. Life satisfaction and happiness associated with depressive symptoms among university students: a cross-sectional study in Korea. Annals of General Psychiatry. 2018;17(1):52. doi: 10.1186/s12991-018-0223-1.

64.	Life satisfaction and risk of depression: The role of adolescents’ social-emotional skills [Internet]. Spain: AITANA Investigación; 2025

65.	Gigantesco A, Fagnani C, Toccaceli V, Stazi MA, Lucidi F, Violani C, et al. The Relationship Between Satisfaction With Life and Depression Symptoms by Gender. Front Psychiatry. 2019;10:419. doi: 10.3389/fpsyt.2019.00419.

66.	Chang Y-H, Yang C-T, Hsieh S. Social support enhances the mediating effect of psychological resilience on the relationship between life satisfaction and depressive symptom severity. Scientific Reports. 2023;13(1):4818. doi: 10.1038/s41598-023-31863-7.

67.	Kwon M, Moon W-h, Kim SA. Mediating effect of life satisfaction and depression on the relationship between cognition and activities of daily living in Korean male older adults. Journal of Men's Health. 2023;19(11):73-81. doi: 10.22514/jomh.2023.118.
