# Supplementary material for: Water, sanitation, and depressive symptoms in Indonesia: The mediating role of life satisfaction
Source: PLoS One. 2026 Feb 5;21(2):e0341886. doi: 10.1371/journal.pone.0341886 (PMC12875457; doi:10.1371/journal.pone.0341886)
Supplement: Attachment — Submitted filename: Response to Reviewer.docx [file pone.0341886.s013.docx]

Manchester, 15 December 2025

To: Dr Alison Parker

Academic Editor, PLOS ONE

Re: Revised Manuscript (PONE-D-25-55524)

Dear Dr Alison,

We sincerely appreciate your interest in our submission to PLOS ONE and are especially grateful for the opportunity to revise and resubmit our manuscript. We are thankful for the valuable and constructive feedback from the reviewers and the editorial team, which has been instrumental in improving our work.

In response to the editorial request regarding funding disclosure, we confirm the following Role of the Funder. This research was funded by the National Institute for Health and Care Research (NIHR) [Global Health Research for Sustainable Care for Anxiety and Depression in Indonesia (Award ID NIHR134638)] using UK international development funding from the UK Government to support global health research. The views expressed in this publication are those of the author(s) and not necessarily those of the NIHR or the UK Government. The funders had no role in the study design, data collection and analysis, decision to publish, or preparation of the manuscript.

To ensure clarity and transparency, we have prepared a detailed, point-by-point response addressing each editor and reviewer’s comments and recommendations. All line numbers referenced in this response correspond to the clean, revised manuscript (without track changes).

We sincerely hope that our revised manuscript now aligns with the journal's standards and criteria, and we earnestly look forward to your favourable consideration for publication in PLOS ONE.

Yours sincerely,

On behalf of the Authors,

Dr. Asri Maharani

**Comments from Editor #1**

Please ensure that your manuscript meets PLOS ONE's style requirements, including those for file naming. The PLOS ONE style templates can be found at

https://journals.plos.org/plosone/s/file?id=wjVg/PLOSOne_formatting_sample_main_body.pdf [journals.plos.org] and

https://journals.plos.org/plosone/s/file?id=ba62/PLOSOne_formatting_sample_title_authors_affiliations.pdf [track.editorialmanager.com]

**Authors’ response**

Thank you for the reminder. We have revised the manuscript and accompanying files to ensure full compliance with PLOS ONE’s formatting and file-naming requirements.

**Comments from Editor #2**

Thank you for stating the following financial disclosure:

“This research was funded by the NIHR [Global Health Research for Sustainable Care for anxiety and depression in Indonesia (Award ID NIHR134638)] using UK international development funding from the UK Government to support global health research. The views expressed in this publication are those of the author(s) and not necessarily those of the NIHR or the UK government. Please state what role the funders took in the study.”

If the funders had no role, please state: "The funders had no role in study design, data collection and analysis, decision to publish, or preparation of the manuscript."

If this statement is not correct you must amend it as needed. Please include this amended Role of Funder statement in your cover letter; we will change the online submission form on your behalf.

**Authors’ response**

Thank you for the clarification. We confirm that the funders had no role in the study design, data collection and analysis, the decision to publish, or the preparation of the manuscript. We have included the required statement in the cover letter as requested. The funder statement thus revised as: “This research was funded by the National Institute for Health and Care Research (NIHR) [Global Health Research for Sustainable Care for anxiety and depression in Indonesia (Award ID NIHR134638)] using UK international development funding from the UK Government to support global health research. The views expressed in this publication are those of the author(s) and not necessarily those of the NIHR or the UK government. The funders had no role in study design, data collection and analysis, decision to publish, or preparation of the manuscript.” We have also ensured that this updated funding and role-of-the-funder statement is reflected in the online submission system.

**Comments from Editor #3**

Please expand the acronym “NIHR” (as indicated in your financial disclosure) so that it states the name of your funders in full.

This information should be included in your cover letter; we will change the online submission form on your behalf.

**Authors’ response**

We have expanded the acronym NIHR to its complete form, *National Institute for Health and Care Research*, in the revised funding statement and cover letter as requested.

**Comments from Editor #4**

Please note that funding information should not appear in any section or other areas of your manuscript. We will only publish funding information present in the Funding Statement section of the online submission form. Please remove any funding-related text from the manuscript.

**Authors’ response**

We have removed all funding-related text from the manuscript, in accordance with the journal’s guidelines.

**Comments from Editor #5**

Your ethics statement should only appear in the Methods section of your manuscript. If your ethics statement is written in any section besides the Methods, please move it to the Methods section and delete it from any other section. Please ensure that your ethics statement is included in your manuscript, as the ethics statement entered into the online submission form will not be published alongside your manuscript.

**Authors’ response**

Thank you for the guidance. We have moved the ethics statement to the Methods section as requested and removed it from all other sections.

**Comments from Editor #6**

If the reviewer comments include a recommendation to cite specific previously published works, please review and evaluate these publications to determine whether they are relevant and should be cited. There is no requirement to cite these works unless the editor has indicated otherwise.

**Authors’ response**

Thank you for the suggestion. We have reviewed all publications recommended by the reviewers and have cited those that are relevant to the scope and analysis of our study, while excluding works that did not align with the manuscript’s focus.

The relevant reviewer-recommended references have been incorporated and are provided below:

Wulandari R, Iswara AP, Qadafi M, Prayogo W, Astuti RDP, Utami RR, et al. Water pollution and sanitation in Indonesia: a review on water quality, health and environmental impacts, management, and future challenges. Environmental Science and Pollution Research. 2024;31(58):65967-92. doi: 10.1007/s11356-024-35567-x.

Fakhrunnisak D, Patria B. The positive effects of parents' education level on children's mental health in Indonesia: a result of longitudinal survey. BMC Public Health. 2022;22(1):949. doi: 10.1186/s12889-022-13380-w.

Marvianto R, Kurniawan D, Husein M. Unveiling the Characteristics of Depression Symptoms in Indonesia Population: Lesson Learned from the 5th Wave of the Indonesian Family Life Survey (IFLS-5). SSRN. 2025. doi: 10.2139/ssrn.5365151.

**Comments from 1^st^ Reviewer #1**

Abstract: Line 31: Please add the duration of observation. How long was the questionnaire data collected?

**Authors’ response**

Thank you for this helpful suggestion. We have now added the IFLS-5 data collection period (September 2014 to March 2015) to both the Abstract and Methods sections.

**Comments from 1^st^ Reviewer #2**

Abstract: Line 36: Correct the numerical error: *(β=0.54)* → *(β=0.054).*

**Authors’ response**

Thank you for pointing out this error. We have corrected the coefficient to **β = 0.054** in the Abstract.

**Comments from 1^st^ Reviewer #3**

Introduction: Lines 63–69: The information presented is relevant to the title. However, paragraphs 2 and 3 appear to lack coherence. The connection between the discussion of depression in Indonesia (paragraph 2) and issues of water and sanitation (paragraph 3) is somewhat abrupt. It would be helpful to include data sources related to population size, water quality vulnerability, and sanitation health in Indonesia.

**Authors’ response**

Thank you for this helpful comment. The transition from the discussion of depressive symptoms to the subsequent section on water and sanitation has been strengthened in the revised manuscript by explicitly framing sanitation as a determinant of mental health and by incorporating nationally representative data to contextualise Indonesia’s current water and sanitation conditions. These include updated SUSENAS 2024 estimates on access to improved drinking water, toilet ownership, and improved sanitation.

**Comments from 1^st^ Reviewer #4**

Introduction: Lines 80–89: Is the explanation of the mediating role of life satisfaction (LS) in the model theoretically robust enough? The authors should clarify why LS was selected as the primary mediator instead of other social factors, such as social support or environmental stress.

**Authors’ response**

Thank you for this valuable comment. We have clarified the theoretical justification for using life satisfaction (LS) as the primary mediator in the introduction. LS is a widely used cognitive indicator of subjective well-being, encompassing multiple dimensions such as psychological functioning, sense of purpose and control, social relationships, and perceived quality of life, and has a well-established conceptual link to both environmental conditions and mental health outcomes. Theoretically, LS reflects an individual’s overall appraisal of their living environment, resources, and daily circumstances, making it a suitable intermediary between household sanitation conditions and depressive symptoms.

Regarding alternative mediators, IFLS-5 does not include a validated measure of social support (e.g., MSPSS or ISEL). The variables available in IFLS, such as social participation and neighbourhood trust, capture dimensions of social capital rather than interpersonal or emotional support and therefore do not align conceptually with the mediating role proposed in our pathway. While some prior IFLS-based studies have incorporated external environmental exposure data (e.g., satellite-derived PM₂․₅ or wildfire smoke estimates), IFLS itself does not collect validated measures of perceived environmental stress, neighbourhood disorder, or climate-related stress that could serve as psychosocial mediators within an SEM pathway. These clarifications have been added to the Methods section.

**Comments from 1^st^ Reviewer #5**

Method: Table 1: For *liquid waste disposal*, the “safe” category lists discharge into rivers, while the “unsafe” category also includes discharge into rivers. The authors should clarify the distinction between these two classifications.

**Authors’ response**

Thank you for the comment. In our original submission, the discharge of wastewater into rivers was mistakenly classified as “safe.” We have now corrected this classification. In the revised manuscript, only drainage systems such as flowing sewers, non-flowing sewers/gutters, and permanent pits are classified as “safe,” as these represent designated household disposal channels. All disposal into natural water bodies, including rivers, ponds, lakes, and the sea, is now classified as “unsafe.”

In addition, following this comment, we rechecked all sanitation variables for consistency and identified that the coding order for the water source variable also required correction. We have revised the coding to ensure accurate categorisation, rerun all analyses, and updated Tables 1 and 3 accordingly. The coding has been updated accordingly, the analyses have been re-run, and Table 1 has been revised to reflect the corrected definitions.

We also used this opportunity to recheck all descriptive tables, correct minor typographical errors, and add missing data where previously omitted to ensure the completeness and accuracy of reporting.

**Comments from 1^st^ Reviewer #6**

Method: Lines 115–134: The explanation of the IFLS dataset is comprehensive; however, the access time (“November 2024”) appears inconsistent with the 2014–2015 data. This should be corrected to avoid the impression that the data were accessed after the analysis had been conducted.

**Authors’ response**

Thank you for this helpful observation. The date we initially provided referred to when the research team accessed the dataset for this project, not to the IFLS data collection period (2014–2015). To avoid confusion and since this information is not essential to understanding the dataset, we have removed the sentence from the revised manuscript. The Methods section now reports only the official IFLS-5 data collection period.

**Comments from 1^st^ Reviewer #7**

Method: Ensure consistent use of “Model 1” and “Model 2” or “Model A” and “Model B.” In the Methods section, the text refers to Models 1 and 2, whereas in the Analysis section, it switches to Models A and B.

**Authors’ response**

Thank you for pointing this out. We have revised the terminology in the Statistical Analysis section to ensure consistency across the manuscript. Specifically, all model has been changed to “Model A” and “Model B” respectively, to match the labels used in the Methods and Results section. In addition, Figure 1 has been revised to reflect these changes, including updating the outcome label from depression to depressive symptoms and explicitly indicating the covariates included in each model, consistent with the SEM specifications described in the Statistical Analysis section.

**Comments from 1^st^ Reviewer #8**

The discussion remains overly descriptive and does not sufficiently emphasise the study’s novelty compared with previous research. The authors could add a concluding paragraph to Section 4.1 highlighting the contribution of this study relative to research conducted in Bangladesh or Ghana.

**Authors’ response**

Thank you for the suggestion. We have added a concluding paragraph to Section 4.1 that highlights the contribution of our findings relative to studies from Bangladesh, Ghana, and similar contexts.

**Comments from 1^st^ Reviewer #9**

Spellings:

1. Lines 231-232: Please use the present tense in the entire result sentence, e.g. "Table 2 presents..."
2. Lines 381-383: The sentence "WHO further estimated that every US$1 invested...", please change to "The WHO further estimates..." in order to parallel the academic style.
3. Use the term “life satisfaction (LS)” consistently throughout the manuscript. Some sections spell it out in full without the abbreviation.
4. Add a source note to each table (e.g., *Data source: IFLS-5, authors’ calculation*).
5. In British style, the use of a comma before *and* is not required unless necessary to prevent ambiguity.
6. Line 274: “Standardized coefficient” 🡪 “Standardised coefficient”
7. Line 384: “sanitation programs…” 🡪 “sanitation programmes…”
8. Please review and ensure consistency with British English conventions across the manuscript.
9. Improve sentence structure for clarity and conciseness, avoiding redundancy.

**Authors’ response**

We have now implemented all recommended revisions. Specifically, we (1) corrected verb tenses in the Results section, (2) revised the sentence beginning “WHO further estimated…” for consistency, (3) standardised the use of “life satisfaction (LS)” across the manuscript, (4) added source notes to all tables, (5) adjusted comma use in accordance with British English conventions, (6) corrected “standardized” to “standardised,” (7) changed “sanitation programs” to “sanitation programmes,” (8) reviewed the manuscript for full consistency with British English spelling, and (9) improved sentence structure throughout for clarity and conciseness.

**Comments from 1^st^ Reviewer #10**

Please use the most recent references to support your research. References to numbers [25], [32], [44], [58], and [59] used over the last 10 years.

In example:

Sakti, B., Wibawa, S., Maharani, A. T., Andhikaputra, G., Savira, M., Putri, A., Iswara, A. P., Sapkota, A., Sharma, A., Syafei, A. D., & Wang, Y. (2023). Effects of Ambient Temperature , Relative Humidity , and Precipitation on Diarrhea Incidence in Surabaya. *International Journal of Environmental Research and Public Health*, *20*, 2313.

Wulandari, R., Iswara, A. P., Qadafi, M., Prayogo, W., Astuti, R. D. P., Utami, R. R., Jayanti, M., Awfa, D., Suryawan, I. W. K., Fitria, L., & Andhikaputra, G. (2024). Water pollution and sanitation in Indonesia: a review on water quality, health and environmental impacts, management, and future challenges. *Environmental Science and Pollution Research*, *0123456789*. https://doi.org/10.1007/s11356-024-35567-x

**Authors’ response**

Thank you for highlighting the importance of using recent references. We have carefully reviewed the citations and updated or supplemented recent references where relevant. For example, we incorporated the recent review by Wulandari et al. (2024) to provide contemporary evidence on water quality and sanitation conditions. However, several references flagged as “older” serve foundational methodological functions and cannot be replaced by more recent studies. Reference (Andresen et al., 1994) provides the original validation of the CES-D–10 scale, which remains the standard reference in both global and Indonesian research. In addition, Amorim et al. (2010) is a core methodological paper that summarises the application of structural equation modelling in epidemiology; more recent SEM literature builds upon, rather than replaces, this foundational work.

Moreover, we have replaced the reference (Guardiola et al., 2014) with a more recent, comparable study (Ammar & Kouser, 2022). Reference (Nelson et al., 2014), which directly examines sanitation conditions and life satisfaction using IFLS data, has been retained because it is uniquely relevant to our study’s focus on WASH factors and subjective well-being in Indonesia. We have therefore updated the references where appropriate and retained essential foundational citations when no more recent or suitable alternatives exist. A list of updated references is reflected in the revised manuscript.

**Comments from 2^nd^ Reviewer #1**

The authors consistently refer to “depression” as the main outcome variable. However, the Indonesian Family Life Survey (IFLS-5) does not include a diagnostic assessment of depression. Instead, it measures depressive symptoms using the Center for Epidemiologic Studies Depression Scale (CES-D-10). This instrument is a screening tool that assesses the frequency and intensity of depressive symptoms, not a clinical diagnosis.

Replace “depression” with “depressive symptoms” throughout the manuscript, including the title, abstract, methods, results, discussion, and conclusion.

**Authors’ response**

We have revised the manuscript to use “depressive symptoms” consistently when referring to our study outcome, which is measured using the CES-D-10 screening instrument. References to “depression” have been retained only when describing findings from other studies that used that terminology.

**Comments from 2^nd^ Reviewer #2**

Abstract: In abstract you mention participants aged >18 and in method > 15 , please check this

**Authors’ response**

Thank you for the comment. The IFLS individual questionnaire is administered to respondents aged 15 years and older, and our analyses therefore include participants aged 15 and older. We have revised the Abstract to ensure consistency with the Methods section.

**Comments from 2^nd^ Reviewer #3**

The secondary data used is outdated (survey results from 10 years ago) and no longer relevant to current circumstances. Therefore, the results cannot be used as a reference for other researchers or as input for developing national health policy in Indonesia. My suggestion is to use data from the 2023 Indonesian Health Survey, as it will be more relevant.

**Authors’ response**

Thank you for the comment. We acknowledge that using recent data is desirable. However, the 2023 Indonesian Health Survey (SKI 2023) does not include the key variables required for our analysis, specifically, life satisfaction, which is needed to build SEM pathways. We thus used the IFLS, a publicly available national dataset in Indonesia that includes the CES-D-10 depression scale, a life-satisfaction measure, detailed household-level sanitation and water variables, and socioeconomic and demographic variables required for SEM. These variables are essential for modelling the direct and indirect pathways in our conceptual framework.

In addition, IFLS remains widely used and is considered current in peer-reviewed research (Renanita and Rembulan, 2025; Jannah et al., 2025; Hilal and Al-Aghbari, 2025; Alfaqeeh et al., 2025). Moreover, our primary research question is the relationship between household water and sanitation conditions, depression, and life satisfaction, rather than estimates of prevalence. While population characteristics and levels of WASH access may change over time, the relationship between WASH conditions and mental health outcomes are likely to be more stable. For example, the 2024 national SUSENAS data show that:

1. Although IFLS-5 was collected in 2014–2015, national WASH trends from SUSENAS 2020–2024 show that conditions have changed only modestly over the past decade. For example, access to improved drinking water increased only slightly nationally. From approximately 86% in IFLS-5 to 92% in SUSENAS 2024, a change of ~6 percentage points over ten years. The distribution of primary water sources also remains comparable: SUSENAS 2024 shows substantial reliance on drilling wells/pumps (43.6%), piped water (9.1%), bottled water (16.5%), and protected springs (9.15%), patterns that align with the IFLS-5 distribution of improved water sources (86.3%).
2. Similarly, access to improved sanitation increased only moderately, from 74% in IFLS-5 to 83% in SUSENAS 2024, a difference of just ~9 percentage points. The proportion of households with a private, non-shared toilet facility in 2024 is 89%, comparable to the IFLS figure when stricter definitions of improved toilet facilities are applied (74%).

Overall, when comparing IFLS-5 with recent SUSENAS statistics, the magnitude of change in national WASH indicators over the past decade is modest, generally within 6–10 percentage points. These patterns indicate that structural disparities in water, sanitation, and waste management persist over time, reinforcing the continued relevance of IFLS-based analyses for understanding the mechanisms linking WASH conditions, depression, and life satisfaction.

**Comments from 2^nd^ Reviewer #4**

Introduction: The introduction is overly long, kindly try to summarize the main points

**Authors’ response**

Thank you for this suggestion. We have revised the introduction by removing redundant statements, tightening the paragraph structure, and streamlining the background information to enhance clarity and conciseness. The main arguments remain intact, but the overall introduction is now more focused and succinct.

**Comments from 2^nd^ Reviewer #5**

Introduction: Can you clarify more about the novelty as the is a previous study almost studied similar variables also using the same data (Water and sanitation quality on depressive symptoms across urban and rural areas in Indonesia: evidence from IFLS-5 data)

**Authors’ response**

We recognise that the previous study using IFLS-5 provides necessary initial evidence on the association between WASH conditions and depressive symptoms. However, our study makes several key methodological and conceptual contributions beyond that work.

First, the previous study relied on descriptive statistics, bivariate tests, logistic regression stratified by rural/urban residence, and a difference-in-coefficients mediation approach, with comorbidity as the mediator. In contrast, our study uses structural equation modelling (SEM), which enables us to estimate direct and indirect pathways simultaneously and to address potential endogeneity among sanitation, life satisfaction (LS), and depression. SEM provides standardised effect sizes, formal model fit indices, and explicitly tests hypothesised pathways rather than relying on sequential regression models.

Second, while the earlier study grouped WASH indicators into a combined model, we analyse each sanitation factor separately in a series of SEM models, thereby isolating the unique contribution of each WASH component. This produces a clearer understanding of which specific sanitation dimensions are most relevant for LS and depression. Third, our study introduces life satisfaction as a mediating variable, which has not been examined before in the context of Indonesian WASH and mental health.

Overall, our work extends prior research by applying a more robust analytical framework, examining distinct sanitation factors, incorporating LS as a mediator, and providing a pathway-oriented understanding of how sanitation conditions relate to depression. These advances offer new insights for mental health and public health policy in Indonesia.

**Comments from 2^nd^ Reviewer #6**

Method: May you make sure about the validity, you can read articles by (Fakhrunnisak & Patria, 2022; Marvianto, 2023).

**Authors’ response**

Thank you for raising the issue of measurement validity. The CES-D-10 has demonstrated good validity and reliability in LMICs, including settings comparable to Indonesia. A psychometric evaluation across 27 LMICs identified a two-factor structure (depressive affect and positive affect) with acceptable internal consistency (α = .77 and α = .57, respectively) and good fit under a graded response model (James et al., 2020). This supports its use as a measure of depressive symptomatology in diverse cultural and socioeconomic contexts. Consistent with prior IFLS-based studies, we classified respondents as experiencing depression at CES-D-10 ≥ 10, which aligns with established cut-offs used internationally and in Indonesian analyses.

Regarding life satisfaction, although IFLS includes a single-item global measure rather than the multi-item SWLS scale, single-item LS measures have strong empirical support. Fonberg & Smith (2019) demonstrated that a single LS question shows robust convergent and discriminant validity when compared with multi-item scales, personality constructs, affect, and anxiety/depression.

In Indonesia, both CES-D-based depressive symptoms and single-item LS measures have also been used consistently and meaningfully in recent psychological and public health studies, further supporting their applicability and relevance in the present analysis (Fakhrunnisak & Patria, 2022; Marvianto, 2023; Renanita, 2025; Choirina, 2021; Pratiwi, 2025). These additions have also been reflected in the manuscripts.

**Comments from 2^nd^ Reviewer #7**

Method: Table 1 Classifications of sanitation conditions. How did you classify them can you supported with references.

**Authors’ response**

We apologise for the earlier lack of clarity. In the revised manuscript, we have expanded the description of how each sanitation indicator was classified and added explicit references supporting these categorisations. The Methods section now clearly explains that our classification follows the WHO/UNICEF Joint Monitoring Programme (JMP) definitions of “improved” versus “unimproved” sanitation and water sources, as well as approaches used in previous IFLS-based sanitation studies (Hilal & Al-Aghbari, 2025); Islam, Rahman, Uddin. et al., 2022)).

**Comments from 2^nd^ Reviewer #8**

Method: The previous study by Mulyaningsih et al. (2023) also introduced composite index to measure access to all component of WASH instead of measuring it separately. The author should explain the advantage of measuring using current measure and the index.

**Authors’ response**

Thank you for the insightful comment. We appreciate that Mulyaningsih et al. (2023) constructed a composite WASH index to summarise overall access to water, sanitation, and hygiene services. Composite indices provide a single aggregated score that simplifies complex phenomena and facilitates comparisons across units, such as countries. However, their reliability depends heavily on how they are constructed, as they collapse multiple components into a single measure (Schlossarek et al., 2019), potentially obscuring the distinct contributions of each sanitation factor.

The present study focuses on examining whether individual sanitation components (e.g., drinking water, toilet facilities, and liquid waste disposal) are associated with depressive symptoms through distinct direct and indirect pathways involving life satisfaction. Modelling each component separately within a structural equation modelling framework enables the estimation of direct, indirect, and total associations, offering clearer insight into the relative contribution of different sanitation dimensions. Moreover, the components of a WASH index may not form a single underlying construct in the context of mental health; combining them assumes homogeneity that may not hold for psychosocial outcomes. Examining each sanitation dimension individually avoids these assumptions and supports more targeted policy interpretation. Additionally, composite indices depend on statistical weighting techniques such as PCA, which are less suitable for pathway-focused modelling. Modelling each sanitation variable separately within SEM, therefore, provides a more precise, theoretically aligned approach to understanding how environmental conditions shape mental health.

**Comments from 2^nd^ Reviewer #9**

Method: Clarify why life satisfaction was selected over other psychosocial variables (e.g., social support) also it is available in IFLS-5.

Mediator measurement: Life satisfaction was measured with a single item, which weakens reliability. Justification is mentioned but not critically discussed.

**Authors’ response**

Thank you for these thoughtful comments.

1. Rationale for selecting life satisfaction over other psychosocial variables.

We agree that psychosocial factors may play an essential role in the pathway between environmental conditions and mental health. However, IFLS-5 does not contain a validated social support scale (e.g., MSPSS, ISEL, SSQ). The variables available, such as social participation and neighbourhood trust, reflect components of social capital rather than interpersonal or emotional support. These items capture civic engagement, community activities, and perceptions of safety and trust, and have been used in previous research as indicators of structural or cognitive social capital rather than as measures of social support.

Because these variables do not represent individual-level psychosocial support and do not align conceptually with our hypothesised pathway, they are not appropriate mediators in our SEM framework. Life satisfaction, by contrast, is a global cognitive appraisal of one’s life conditions and is theoretically positioned between environmental exposures and depressive symptoms. For these reasons, LS was selected as the most suitable psychosocial mediator available in IFLS-5.

2. Use of a single-item LS measure.

While single-item measures may not fully capture the multidimensional nature of quality of life, they have been shown to demonstrate strong convergent and discriminant validity when compared with multi-item life satisfaction scales and related psychological constructs. Fonberg & Smith (2019) showed that single-item LS measures correlate highly with multi-item scales and with broader indicators such as affect, optimism, and anxiety/depression, supporting their suitability for population-level research. Importantly, several peer-reviewed Indonesian studies using IFLS data have also relied on the same LS item and have demonstrated consistent and meaningful associations with mental health outcomes [39–41]. We have expanded the Methods section (lines 203-221) to clarify the conceptual reasons for selecting LS and to strengthen the justification for the single-item measure.

**Comments from 2^nd^ Reviewer #10**

Method: Consider sensitivity analyses including key socioeconomic covariates, even if model fit worsens. Explain about missing data handling strategy.

**Authors’ response**

Thank you for the suggestion. We have conducted additional sensitivity analyses by including all key socioeconomic covariates (education level, occupation status, residency type, and wealth) in the sanitation variables. As expected, model fit worsened substantially when these variables were added. The full fit indices of this sensitivity analysis are reported in Table S3.

These results indicate that including all socioeconomic variables simultaneously leads to poor model fit, particularly for drinking water, water source, and toilet facilities, supporting our decision to retain only occupation status and residency type in the final SEM models, where fit remained acceptable. This aligns with standard SEM practice, prioritising model stability and theoretical coherence.

Regarding missing data, we have clarified our handling method in the revised manuscript. Missing data across all variables was minimal, ranging from 0.01% to 6.58%. Since several important variables, such as sanitation classifications, chronic disease count, LS, and depressive symptoms, are categorical and conceptually specific, we did not impute these variables. Instead, we used listwise deletion, which is appropriate when missingness is low and imputation could introduce bias (Little and Rubin, 2019). An explanatory statement has been included in the Methods section.

**Comments from 2^nd^ Reviewer #11**

Figure 1. Provide a path diagram with standardized coefficients for transparency.

**Authors’ response**

Thank you for the suggestion. Figure 1 is intended to present the conceptual pathways underlying our analysis rather than to display statistical estimates. To maintain clarity and avoid the need for multiple figures, we have retained the diagram as a conceptual illustration only. All SEM results, including standardised coefficients and 95% confidence intervals for every pathway, are already fully reported in Table 3, which provides a transparent and comprehensive presentation of the findings.

**Comments from 2^nd^ Reviewer #12**

Results: Although sampling weights are mentioned, it is not clear if SEM analyses were weighted.

**Authors’ response**

Thank you for the comment. This approach is consistent with prior IFLS-based SEM studies (which typically do not incorporate survey weights) and with established methodological guidance for analyses focused on modeling theoretical associations and pathways. When the primary objective is to examine relationships between variables rather than to produce precise population-level descriptive estimates, sampling weights may not be necessary and can sometimes reduce estimator efficiency. We have included the following studies using IFLS-5 and SEM as the statistical analysis method:

Renanita T, Rembulan CL. The Effects of Social Comparison and Subjective Financial Well-Being on Subjective Well-Being. Europe’s Journal of Psychology. 2025;21(3):179-93. doi: 10.5964/ejop.14791.

Bakar A, Diana Fitri YA, Afriza D, Arma U, Darmawangsa D, Ningrum V. Structural Equation Modeling of Oral Stomatitis and Its Determinants among the Sundanese Ethnic Group: Evidence from IFLS-5. International Journal of Statistics in Medical Research. 2025;14:355-63. doi: 10.6000/1929-6029.2025.14.34.

And a theoretical reference:

Bollen, K. A., Biemer, P. P., Karr, A. F., Tueller, S., & Berzofsky, M. E. (2016). Are survey weights needed? A review of diagnostic tests in regression analysis. Annual Review of Statistics and Its Application, 3, 375–392. https://doi.org/10.1146/annurev-statistics-011516-012958

**Comments from 2^nd^ Reviewer #13**

Discussion: Rephrase causal statements to reflect association only

**Authors’ response**

Thank you for the comment. We have thoroughly reviewed the manuscript and revised all instances of causal phrasing to ensure that associations are described appropriately. Terms such as “predicts” have been removed or replaced with non-causal wording (e.g., “associated with” or “linked to”) throughout the Discussion and Conclusion.

**Comments from 2^nd^ Reviewer #14**

4.4 Strengths, limitations, and directions for future research: Add limitations about the single-item LS measure and the outdated data wave.

**Authors’ response**

We have added the requested limitations regarding the use of a single-item life satisfaction measure and the age of the IFLS-5 dataset. These points have been incorporated into the Strengths and Limitations section.

**Comments from 2^nd^ Reviewer #15**

Conclusion: Rephrase causal language — replace “predictors” with “associated factors” or “correlates”.

**Authors’ response**

Thank you for the comment. We have removed causal wording in the text and replaced “predictors” with non-causal terms such as “associated factors” or “correlates” where appropriate.

**Comments from 2^nd^ Reviewer #16**

Ethic Statement: May you add the EC No

**Authors’ response**

We have now added the EC number to the Ethics Statement, lines 268-279.

**Comments from 3^rd^ Reviewer #1**

Abstract: Ensure consistency in reported β values (e.g., some typos: water source β=0.54 likely should be 0.054).

**Authors’ response**

Thank you for nothing for this typo. We have ensured all numbers reported are consistent throughout the manuscript.

**Comments from 3^rd^ Reviewer #2**

Abstract: Add a sentence summarizing the policy implication at the end of the abstract (e.g., “Findings underscore the need to integrate WASH improvements into national mental health policies.”)

**Authors’ response**

Thank you for the suggestion. We have added a concluding sentence to the abstract to summarise the policy implications, noting the importance of integrating WASH improvements into national mental health and public health strategies.

**Comments from 3^rd^ Reviewer #3**

*“In Indonesia, where disparities in access to clean water and sanitation facilities remain evident, with approximately 47% of the population lacking adequate sanitation and 22% lacking access to clean water…”*

Introduction: Please update the cited percentages to reflect the latest SUSENAS data (e.g., 2022–2023 or 2024) and specify the year of the dataset. This will strengthen the accuracy and contemporary relevance of the background section. If older data are intentionally used, please clarify the rationale for doing so.

**Authors’ response**

Thank you for this helpful suggestion. The sentence referring to outdated percentages has been removed. In its place, we have added a new paragraph summarising the most recent national estimates based on SUSENAS 2024 data (Lines 64-75), which provide up-to-date statistics on access to clean water and sanitation in Indonesia. This revision strengthens the accuracy and contemporary relevance of the background section.

**Comments from 3^rd^ Reviewer #4**

*“Such research is relevant where environmental inequities and vulnerabilities intersect, but empirical evidence linking infrastructure to psychological outcomes is scarce…”*

Introduction: This statement makes two substantive claims: (1) that environmental inequities intersect with health vulnerabilities, and (2) that empirical evidence linking infrastructure (e.g., water, sanitation) to psychological outcomes is limited. Please provide supporting references to strengthen this argument.

**Authors’ response**

Thank you for this helpful suggestion. We have revised the paragraph to more clearly connect Indonesia’s elevated mental health needs (paragraph 2, lines 55-63) with existing environmental constraints (paragraph 3, lines 64-75) and to improve the transition to the study aim. We also added wording that clarifies why this intersection makes Indonesia an essential context for investigating water, sanitation, and psychological outcomes. The revised sentence now reads:

“In Indonesia, where disparities in access to clean water and sanitation facilities remain evident [8, 30], investigating the potential impact of these issues on mental health is essential. Such research is particularly relevant in settings where elevated mental health needs coincide with environmental constraints, yet empirical evidence linking basic infrastructure to psychological outcomes remains scarce.”

Added references:

BPS-Statistics Indonesia. Statistical Yearbook of Indonesia 2025. Badan Pusat Statistik/BPS-Statistics Indonesia, 2025.

Afifah T, Nuryetty MT, Cahyorini, Musadad DA, Schlotheuber A, Bergen N, et al. Subnational regional inequality in access to improved drinking water and sanitation in Indonesia: results from the 2015 Indonesian National Socioeconomic Survey (SUSENAS). Glob Health Action. 2018;11(sup1). doi: 10.1080/16549716.2018.1496972.

**Comments from 3^rd^ Reviewer #5**

*“Beyond access to sanitation, cultural beliefs significantly influence sanitation behaviours…”*

This part discussing cultural beliefs and sanitation (e.g., supernatural beliefs in rural Bali influencing toilet use) is interesting and relevant, but the transition to the study aim is abrupt. The current text *“Beyond access to sanitation, cultural beliefs significantly influence sanitation behaviours… Given the intertwined environmental, cultural, and social dimensions…”* would benefit from clearer bridging and a more explicit rationale for why cultural factors matter for a study focused on water, sanitation, depression, and life satisfaction.

I recommend revising this section to:
(1) clearly connect cultural influences to psychological and behavioral aspects of sanitation,
(2) explain how these cultural contexts make Indonesia a particularly important case to study, and
(3) lead more naturally to the stated research aim.

A more integrated explanation will strengthen coherence and help readers understand why the cultural dimension is highlighted in the background.

**Authors’ response**

Thank you for this constructive suggestion. We have revised the paragraph to (1) more clearly link cultural beliefs to the psychological and behavioural dimensions of sanitation, (2) explain why Indonesia’s cultural context makes it a particularly important setting for this research, and (3) provide a smoother transition to the study aim. The revised text now reads:

“…Beyond access to sanitation, cultural beliefs significantly influence sanitation behaviours. For example, a study in rural Bali found that supernatural beliefs, perceptions of toilets as polluting, and prioritising religious spending over sanitation investments influenced whether households adopted or used toilets [33]. These culturally rooted practices affect not only sanitation behaviour but also feelings of dignity, social acceptance, and perceived purity, psychological dimensions that influence how individuals experience their environment and may impact overall well-being [34]. Given Indonesia’s distinctive combination of environmental constraints and culturally shaped sanitation practices, this study provides a contextually grounded examination of how water and sanitation relate to depressive symptoms, with LS as a potential mediator.”

**Comments from 3^rd^ Reviewer #6**

*“…this study aims to investigate the relationships between water, sanitation, and depression, with life satisfaction as a potential mediator in the Indonesian context.”*

Add one paragraph at the end of the Introduction stating the study’s novelty clearly

**Authors’ response**

Thank you for the suggestion. We have added a concise paragraph at the end of the Introduction that clearly outlines the study's novelty. This paragraph highlights (1) our use of SEM to examine direct and indirect pathways, (2) our decision to analyse each sanitation component separately rather than using a composite index, and (3) the inclusion of life satisfaction as a mediator, which has not been examined in previous Indonesian studies.

**Comments from 3^rd^ Reviewer #7**

IFLS-5 data were collected in 2014–2015. While this is a high-quality and widely used dataset, the Methods section should provide a clearer rationale for using data that are now a decade old. As sanitation access and mental health awareness in Indonesia have improved in recent years, it is important to explicitly address why IFLS-5 remains appropriate for analysing the *relationships* among water, sanitation, life satisfaction, and depression. I suggest adding a brief explanation here to clarify that:

(1) IFLS-5 is one of the few nationally representative datasets that includes detailed measures of sanitation infrastructure *and* psychological outcomes;

(2) the objective of the study is to examine associations, which are conceptually more stable over time even if prevalence levels have changed; and

(3) the age of the dataset is further acknowledged in the Limitations section.

**Authors’ response**

Thank you for this critical comment. We have added a brief rationale in the 2.1 Data and study population section to clarify the appropriateness of using IFLS-5 despite its age. Specifically, we note that (1) IFLS-5 is one of the few nationally representative datasets in Indonesia that contains both detailed WASH measures and validated psychological outcomes, including life satisfaction; (2) we aim to examine associations, which are conceptually more stable over time even if prevalence has shifted; and (3) the age of the dataset is explicitly acknowledged in the Limitations section.

**Comments from 3^rd^ Reviewer #8**

Covariates: You mention removing wealth and education due to poor model fit. Consider reporting the model fit indices in the supplement (S3 Table) and briefly stating the threshold values used (e.g., RMSEA<0.05, CFI>0.90).

**Authors’ response**

Thank you for the suggestion. We have now added the threshold values used to evaluate model fit (e.g., RMSEA < 0.06, CFI > 0.95, SRMR ≤ 0.08, TLI ≥ 0.95) in the Methods section. In addition, we have reported the model fit indices for the models including all key socioeconomic variables in Supplementary Table S3. When wealth and education were included as additional covariates, several SEM models failed to meet the threshold for acceptable fit. Only the waste disposal model met all thresholds, while others showed suboptimal CFI and TLI values despite adequate RMSEA and SRMR.

**Comments from 3^rd^ Reviewer #9**

Statistical Analysis: Please specify the software used

**Authors’ response**

We have added the software information in the Statistical Analysis section. All analyses were conducted using STATA17.

**Comments from 3^rd^ Reviewer #10**

Statistical analysis: When mentioning good model fit, cite reference cutoffs for SRMR, RMSEA, and CFI.

**Authors’ response**

Thank you for the suggestion. We have added reference cutoffs for model fit indices following Kline (2016). According to Kline’s recommended combination rule, CFI ≥0.95 together with SRMR ≤0.08 indicates an acceptable fit. RMSEA values close to or below 0.06 are typically considered good, whereas upper confidence interval bounds above 0.10 indicate that a poor fit cannot be rejected. These reference cutoffs have now been included in the Statistical Analysis section.

Reference:

Kline RB. Principles and practice of structural equation modeling, 4th ed. New York, NY, US: The Guilford Press; 2016. 273-9 p.

**Comments from 3^rd^ Reviewer #11**

Results: The mean age of 37.32 years (±14.93) is plausible but requires clarification. Since the sample includes adults aged ≥15 years, the authors should state whether this mean is weighted or unweighted, whether any age groups were excluded due to missing data, and the full age range of the analytic sample. This will help ensure the age distribution accurately reflects the IFLS-5 population.

**Authors’ response**

Thank you for the comment. We have clarified the age distribution in the revised manuscript. The mean age of 37.32 years (±14.93) is unweighted. No age groups were excluded beyond cases removed due to missing data on depressive symptoms or chronic disease count. The final analytic sample includes respondents aged 15 to 101 years, which aligns with the age distribution of adults interviewed in IFLS-5. We have added this information to the Results section for clarity.

**Comments from 3^rd^ Reviewer #12**

Theoretical and practical implications: This section would benefit from a deeper and more updated discussion. The theoretical implications should more clearly highlight the study’s novelty. Particularly how examining life satisfaction as a mediator contributes to existing WASH and mental health literature in Indonesia and LMICs. Additionally, the practical implications should be strengthened by linking the findings to current national conditions, such as ongoing disparities in safely managed sanitation, recent policy initiatives (e.g., PPSP, STBM), and Indonesia’s efforts toward SDG 6 and universal access to improved WASH services. Expanding this section would enhance the relevance, timeliness, and contribution of the study.

**Authors’ response**

Thank you for this helpful suggestion. We have revised the Theoretical and Practical Implications section to more clearly articulate the study’s novelty and its relevance to Indonesia’s current water and sanitation conditions. Specifically, we strengthened the theoretical contribution by emphasising the limited empirical research in Indonesia and other LMICs that examines psychosocial pathways between sanitation and mental health, and by highlighting the value of modelling sanitation components separately using SEM. We also expanded the practical implications to link the findings to national priorities, including persistent disparities in safely managed sanitation, ongoing programmes such as PPSP and STBM, and Indonesia’s progress toward SDG 6. These revisions enhance the clarity, relevance, and contribution of the study.

**Comments from 3^rd^ Reviewer #13**

Strengths, limitations, and directions for future research: You mention missing data but not the handling method (listwise deletion, imputation?). Add one line to specify.

**Authors’ response**

Thank you for pointing this out. We have clarified our handling of missing data in the revised manuscript. Missingness across all variables was low (ranging from 0.01% to 6.58%). Because several key variables, including sanitation classifications, chronic disease count, LS, and depressive symptoms, cannot be reliably imputed due to their categorical structure and underlying conceptual meaning, we applied listwise deletion. This approach is now specified in the **Methods section**, and a brief acknowledgement of this handling has also been added to the **Limitations section**, as suggested.
